# Evaluation of a therapeutic education program for patients with coronary heart disease in a Tunisian university hospital: A quasi-experimental study

**DOI:** 10.1371/journal.pone.0361814

**Published:** 2026-11-09

**Authors:** Hela Ghali, Mohamed Ayoub Tlili, Aymen El Hraiech, Jihen Sahli, Sana Bhiri, Saoussen Layouni, Hend Ben Souda, Majdi Karray, Bruno Pavy, Asma Ben Cheikh, Chekib Zedini

**Affiliations:** 1 Department of Preventive and Community Medicine, Sahloul University Hospital, Sousse, Tunisia; 2 Faculty of Medicine of Sousse, University of Sousse, Sousse, Tunisia; 3 Nursing Administration Department, College of Nursing, University of Hail, Hail, Saudi Arabia; 4 Department of Cardiology, Sahloul University Hospital, Sousse, Tunisia; 5 Department of Family and Community Medicine, Sousse, Tunisia; 6 Department of Physical Rehabilitation, Sahloul University Hospital, Sousse, Tunisia; 7 Family Medicine, Faculty of Medicine of Sousse, University of Sousse, Sousse, Tunisia; 8 Faculty of Pharmacy of Monastir, University of Monastir, Monastir, Tunisia; 9 Cardiac Rehabilitation Department, Loire-Vendée-Océan Hospital Center, Machecoul, France; UN Mehta Institute of Cardiology and Research Center, INDIA

## Abstract

**Background:**

Cardiovascular disease (CVD) is a major public health concern globally, including Tunisia. Therapeutic Patient Education (TPE) plays a crucial role in managing CVD and improving patient outcomes. This study aimed to evaluate the impact of a TPE program on the occurrence of major cardiovascular events in patients with coronary heart disease (CHD) in a Tunisian university hospital.

**Methods:**

A quasi-experimental study was conducted over one year, involving 129 patients with CHD. The intervention group (n = 38) received a four-stage TPE program, while the control group (n = 91) received standard care. The control group met the same inclusion and non-inclusion criteria as the intervention group. It was constituted based on a 3:1 ratio. Patients in the intervention and control groups were recruited from the same department and during the same period to ensure comparability. The primary outcome was the occurrence of major cardiovascular events within one year. Secondary outcomes included knowledge, lipid profile, glycated hemoglobin levels, and quality of life.

**Results:**

The occurrence of major cardiovascular events was significantly lower in the intervention group compared to the control group (21.1% vs. 59.3%, p < 0.001). The TPE program was also associated with improvements in knowledge and biological parameters. Knowledge of cardiovascular risk factors increased from 71.1% at baseline to 78.9% immediately post-intervention (p = 0.039), with 60.5% retention at 3 months. The intervention group showed marked improvements in lipid profiles, with LDL cholesterol decreasing from 2.53 ± 1.45 to 1.88 ± 1.11 mmol/L (p < 0.001) and total cholesterol from 4.32 ± 1.47 to 3.75 ± 1.56 mmol/L (p = 0.002) at 3 months. In contrast, the control group demonstrated worsening lipid parameters (LDL: 2.50 ± 1.55 to 2.90 ± 1.60 mmol/L, p = 0.001).

**Conclusions:**

The TPE program was associated with a lower occurrence of major cardiovascular events and improvements in patients’ knowledge and lipid profiles. These findings suggest a potential beneficial effect of structured therapeutic education in patients with CHD.

**Trial registration:**

The study was prospectively registered on the Pan African Clinical Trial Registry within the number PACTR202307694422939. The authors confirm that all ongoing and related trials for this intervention are registered.

## Introduction

Cardiovascular diseases (CVD), including coronary heart disease (CHD), hypertension, stroke, and congenital heart defects, remain a leading cause of morbidity and mortality worldwide [1]. Risk factors such as smoking, unhealthy diet, physical inactivity, and stress contribute significantly to their development. With the global prevalence of CVD rising, there is an increasing need for effective strategies to predict and prevent cardiovascular events [1–3].

CHD alone affected 154 million people in 2016, accounting for 32.7% of the global cardiovascular disease burden [4]. In adults over 20 years, nearly half have some form of CVD [5].

In the Middle East and North Africa, the burden remains particularly high [6]. Specifically in Tunisia, CHD-related mortality increased by 30% between 1997 and 2009, with ischemic heart disease causing 176.5 deaths per 100,000 inhabitants in 2019 [6,7]. This is largely driven by high rates of cardiovascular risk factors, including hypertension (50.4%), dyslipidemia (44%), obesity (31.4%), smoking (24.4%), and diabetes (18.2%) in 2020 [8,9].

Effective management of these risk factors, alongside pharmacological and interventional therapies, is essential to reduce recurrent hospitalizations, cardiovascular complications, and healthcare costs [10–12]. Therapeutic patient education (TPE) has emerged as a key non-pharmacological intervention, empowering patients with the knowledge and skills to manage chronic diseases, improve adherence, and enhance quality of life (QoL) [13]. Evidence shows that educated patients achieve better disease control, fewer complications, and improved outcomes [14,15].

In CHD, TPE supports risk factor control, treatment adherence, and patient confidence, ultimately promoting better prognosis. However, despite its demonstrated benefits in chronic diseases, there is limited evidence on the effectiveness of structured TPE programs specifically targeting patients with coronary heart disease in low- and middle-income countries, particularly in North Africa. Few studies have explored its long-term impact on cardiovascular outcomes, such as the reduction of major cardiovascular events or the maintenance of improved risk factor control over time [11–13].

Therefore, this study aimed to evaluate the effectiveness of a structured TPE program in improving knowledge and QoL, controlling lipid parameters, and reducing the occurrence of major cardiovascular events among patients with CHD.

## Methods

### Study design and setting

This is a quasi-experimental study (Figs 1–2) conducted in the cardiology department of Sahloul University Hospital (Sousse, Tunisia) between May 2022 and August 2023. The design was selected to evaluate the TPE program under real-world clinical conditions.

**Fig 1:**
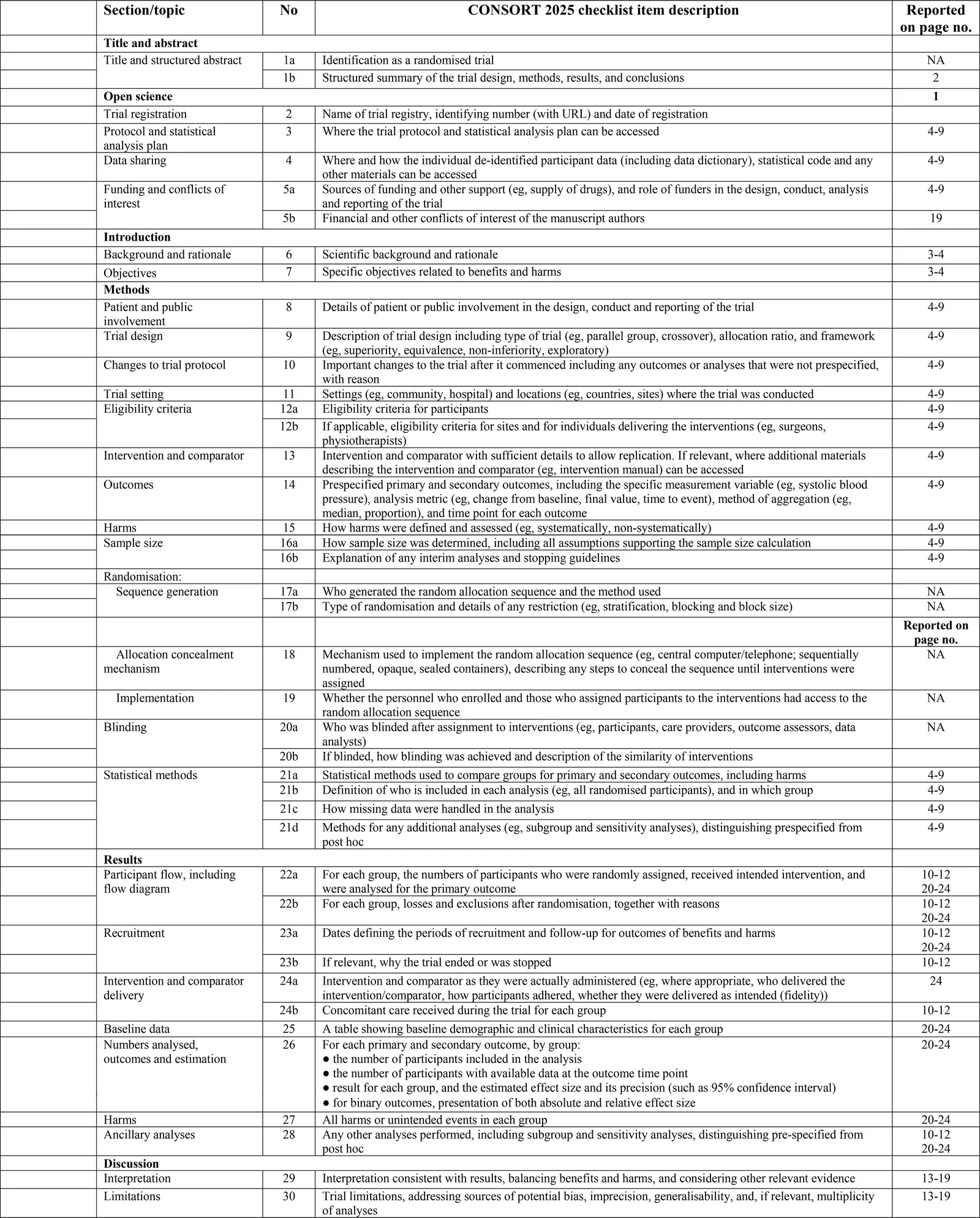
CONSORT flowchart.

**Fig 2:**
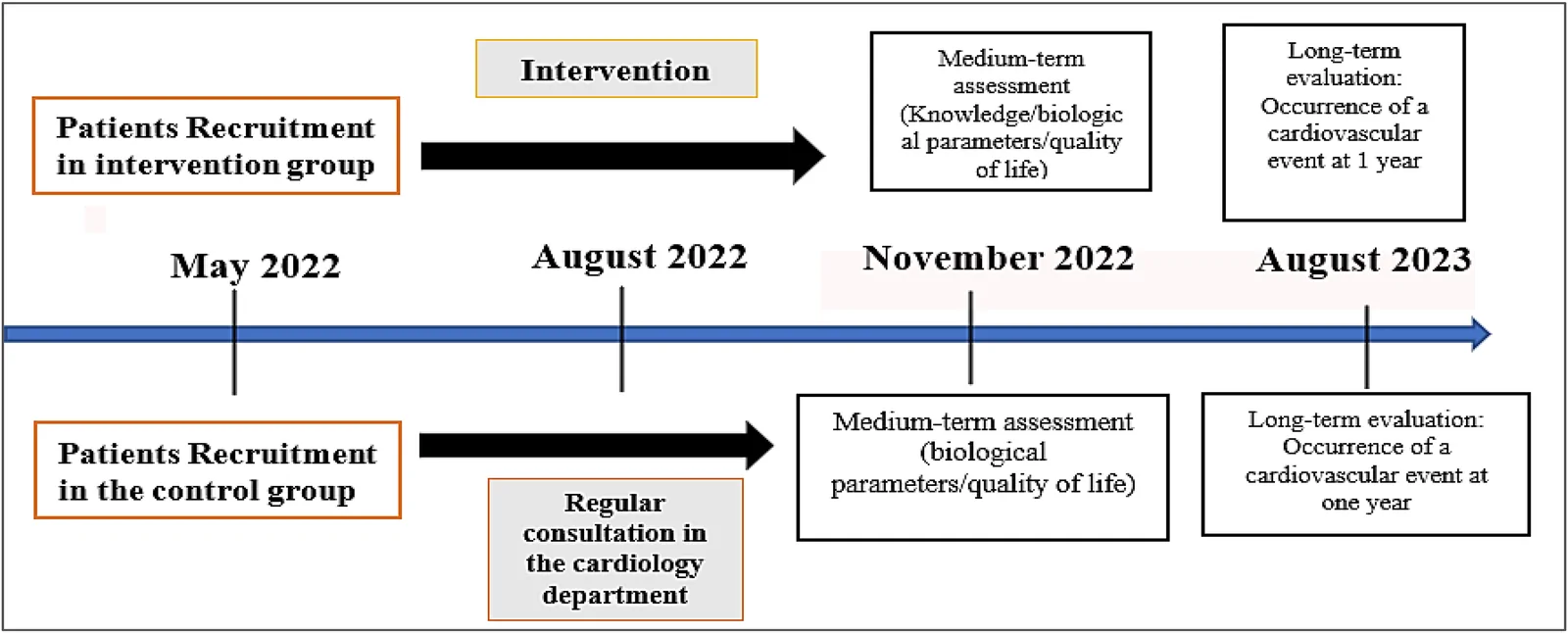
Study timeline.

### Study population and sample size calculation

Participants were consecutively recruited between May 1 and May 31, 2022, and were followed for one year. Eligible patients were adults aged 18 years or older with documented coronary heart disease, either revascularized or awaiting revascularization. The study excluded patients who had substantial cognitive impairment, were hospitalized for non-coronary diseases, or declined to participate in the TPE program.

To reduce selection bias, patients in both the intervention and control groups were recruited from the same department, during the same period, and according to identical inclusion and non-inclusion criteria. Inclusion in the intervention group required informed consent. Patients who declined participation were not included in the study and were not reassigned to the control group.

The sample size was calculated based on parameters reported by Anderson et al. (2017) [16], who demonstrated a significant reduction in cardiovascular events following patient education. The calculation was based on the expected difference in cardiovascular event rates between the intervention and control groups, with a minimum of 37 participants required per group to achieve 80% power at a 5% significance level. Considering the pragmatic nature of the study and the feasibility constraints related to implementing the therapeutic education program in a real-world clinical setting, a 3:1 control-to-intervention allocation ratio was adopted, allowing the inclusion of a larger comparison group while maintaining an adequate number of patients receiving the intervention. Accounting for potential losses to follow-up, a minimum total sample size of 111 participants was considered necessary. Ultimately, 129 participants were enrolled, including 38 in the intervention group and 91 in the control group, providing adequate statistical power for the primary outcome analysis and increasing the robustness of the findings.

### Data collection and outcome measures

Data were collected using a structured questionnaire capturing sociodemographic variables, cardiovascular risk factors, and relevant medical history. Biological parameters, including total cholesterol, HDL cholesterol, LDL cholesterol, triglycerides, and glycated hemoglobin (HbA1c), were measured using routine standardized laboratory methods in the central clinical laboratory of Sahloul University Hospital. Blood samples were collected after an overnight fast of at least 8 hours for lipid profile assessment, while HbA1c was measured irrespective of fasting status according to standard laboratory practice. The hospital laboratory follows established internal quality control procedures. Baseline blood samples were collected before initiation of the therapeutic patient education (TPE) program in the intervention group and during the initial assessment in the control group. Follow-up biological measurements were obtained three months after completion of the intervention for the intervention group and after an equivalent follow-up period for the control group. Participants’ disease-related knowledge was assessed through questions covering coronary heart disease mechanisms, risk factors, symptoms, treatment, diet, and physical activity.

The primary outcome was the occurrence of cardiovascular events within one year of hospital discharge for the control group and within one year of completing the TPE program for the intervention group. Events included acute myocardial infarction, stable angina, coronary revascularization, stroke, hospitalization for heart failure, or cardiovascular-related death. Outcomes were assessed at 12 months through structured follow-up interviews and verified using medical records; information on deaths was obtained from families or healthcare providers.

Secondary outcomes included changes in disease-related knowledge, lipid profile, and glycated hemoglobin levels, as well as changes in health-related QoL. The latter was assessed using the validated Short Form-12 Health Survey (SF-12), which evaluates eight domains: physical functioning, role physical, bodily pain, general health, vitality, social functioning, role emotional, and mental health [17]. Each item was scored according to the standardized algorithm proposed by Ware et al. (1996) [18], with raw domain scores converted to a 0–100 scale, where higher scores reflect better perceived health status. Two composite scores were then derived: the Physical Component Summary (PCS) and the Mental Component Summary (MCS), both standardized to a mean of 50 (SD = 10) in the general population.

The SF-12 was administered once, at the 3-month follow-up, to both groups in order to compare health-related QoL between the intervention and control arms. The SF-12 was selected as a secondary outcome because it offers a concise yet sensitive measure of physical and mental well-being and is widely validated in cardiac populations.

### Intervention (Fig 2)

The TPE program was conducted after hospital discharge, ensuring that intervention and control groups did not interact and minimizing the risk of contamination. The intervention was delivered by healthcare professionals trained in therapeutic patient education (TPE), all of whom had obtained a Complementary Certificate in Therapeutic Patient Education. Before the beginning of the study, educators received specific training on the intervention protocol, including the educational objectives, content, materials, and delivery procedures, to ensure standardization and consistency of the program. The intervention followed a structured four-stage methodology.

### Educational diagnosis

An initial 17-item questionnaire was developed to assess patients’ knowledge of coronary heart disease, including its risk factors, diagnostic procedures, warning symptoms, treatments, and lifestyle recommendations. The questionnaire was designed based on the educational objectives of the therapeutic patient education (TPE) program and current clinical recommendations to evaluate the key concepts addressed during the intervention. Each correct response was awarded one point, with higher scores indicating greater knowledge. Patients were then stratified into five subgroups according to their highest completed level of formal education (primary school, middle school, secondary school, baccalaureate, and higher education). Although all participants received the same educational objectives and core content, the teaching strategies were adapted to each subgroup by adjusting the complexity of the language, the pace of the sessions, and the educational materials. Participants with lower educational levels received simplified explanations supported by visual aids, practical demonstrations, and interactive discussions, whereas those with higher educational attainment were provided with more detailed explanations and encouraged to engage in in-depth discussions. These adaptations aimed to optimize comprehension while maintaining a standardized educational curriculum across all groups. The same questionnaire was administered immediately after the intervention to assess knowledge acquisition and again at three months to evaluate knowledge retention. Although the questionnaire was specifically developed for this study, it did not undergo formal psychometric validation prior to its use.

### Educational contract

Findings from the diagnostic phase guided the development of individualized educational contracts. These contracts specified the competencies to be acquired, knowledge, self-care actions, and appropriate reactions to warning signs, tailored to each patient’s priorities. Objectives included medication monitoring, symptom recognition, physical activity, diet, and lifestyle changes, all defined to be realistic and achievable.

### The TPE program

The therapeutic patient education (TPE) program was developed by a multidisciplinary team including specialists in preventive and community medicine, cardiology, and family medicine. The program was designed in accordance with the World Health Organization (WHO) recommendations on therapeutic patient education and current European Society of Cardiology (ESC) guidelines for the secondary prevention and management of coronary heart disease. The educational objectives and workshop content were established through expert consensus based on the educational needs identified during the initial educational diagnosis. Educational materials, including PowerPoint presentations, Arabic-language summary leaflets, videos, role-playing exercises, card games, and practical workshops, were developed locally by the study team to ensure cultural and linguistic appropriateness for Tunisian patients while remaining consistent with evidence-based recommendations.

The intervention consisted of three weekly sessions, each including three workshops lasting approximately 30–40 minutes and combining theoretical presentations, group discussions, and practical exercises.

The first session provided an overview of coronary heart disease, followed by a video-supported and role-playing workshop on recognizing and responding to chest pain, and a round-table discussion on cardioprotective nutrition and balanced dietary practices.

The second session focused on cardiovascular risk factors and therapeutic targets and included a workshop on interpreting clinical and laboratory examinations, a card-based exercise on the Mediterranean diet, and a discussion on healthy lifestyle recommendations, including smoking cessation, stress management, and physical activity.

The third session addressed pharmacological treatment through a card-based workshop on medication names and indications, provided information on the social security and healthcare reimbursement system, and concluded with a discussion reviewing the knowledge and competencies acquired throughout the program.

Arabic-language summary leaflets were distributed to all participants to reinforce the educational messages.

Finally, each participant attended a brief individual interview during which recommendations were further tailored to their specific needs. All participants assigned to the intervention group attended the complete educational program.

### Evaluation of skills and program progress

At program completion, patients completed a satisfaction questionnaire assessing organization, clarity, duration, responsiveness, and educational material, and provided a global score out of 10.

Control-group participants received standard cardiology care according to routine clinical practice, including regular cardiology consultations, medical management as clinically indicated, and general verbal counseling regarding cardiovascular health, lifestyle recommendations, and treatment adherence. However, they did not receive any structured therapeutic education intervention.

### Statistical analysis

SPSS version 26 was used to analyze the data. Frequencies and percentages were used to characterize qualitative data. Quantitative variables were described according to their distribution as mean ± standard deviation (SD) for normally distributed data or as median [interquartile range (IQR)] for non-normally distributed data. The Student’s *t*-test for normally distributed variables and the Mann-Whitney *U* test for non-normally distributed variables were used for between-group comparisons. The paired *t*-test was used to assess within-group differences over time. McNemar’s test or Pearson’s chi-squared test were used to compare categorical variables. When comparing repeated categorical measurements, the Cochran’s Q test was employed. To determine the magnitude of within-group changes, effect sizes (Cohen’s *d*) with their corresponding 95% confidence intervals (95% CIs) were calculated. For the primary outcome, relative risks (RRs) with 95% confidence intervals (95% CIs) were calculated. All statistical analyses were performed using a two-sided significance level of p < 0.05.

### Ethical considerations

Ethical approval was obtained from the Sahloul University Hospital Ethics Committee (HS12–2022), and the study was registered on the Pan African Clinical Trial Registry (PACTR202307694422939). All participants received clear information about the study and provided written informed consent.

Participant privacy and data protection were rigorously ensured. Identifiable information was removed from all datasets and replaced with coded identifiers. All analyses were conducted on anonymized data, and results were reported in aggregated form to prevent any identification.

Participants were assured that their information would remain confidential and that they could withdraw at any time without affecting their care.

## Results

### Study population (Table 1)

Our study included 129 patients, with 38 (29.4%) belonging to the intervention group and 91 (70.5%) patients included in the control group. The median age was 63.5 years [55.7–69], compared with 66 [58–72] of patients in the control group, with no statistically significant difference (p = 0.259). Participants characteristics are presented in Table 1.

**Table 1:** Socio-demographic characteristics of the study population.

	Group		P-value*
	Intervention (N = 38) n (%)	Control (N = 91) n (%)	
**Gender**			
Male	33 (86.8)	74 (81.3)	0.447
Female	5 (13.2)	17 (18.7)	
Sex-ratio	6.6	4.35	
**Professional status**			
Employed	15 (39.5)	24 (26.4)	0.313
Retired	13 (34.2)	41 (54.1)	
Unemployed	10 (26.3)	26 (28.6)	
**Level of education**			
Primary	15 (39.5)	41 (45.1)	0.082
College	4 (10.5)	20 (22)	
Secondary	11 (28.9)	23 (25.3)	
Baccalaureate	4 (10.5)	5 (5.5)	
Beyond Baccalaureate	4 (10.5)	2 (2.2)	
**History**			
Smoking	28 (73.7)	58 (63.7)	0.275
Hypertension	19 (50)	50 (54.9)	0.608
Stroke	2 (5.3)	10 (11)	0.307
Diabetes mellitus	17 (44.7)	42 (46.2)	0.883
Respiratory disease	0 (0)	3 (3.3)	0.555
Arteritis of the lower limbs	2 (5.3)	2 (2.2)	0.581
Endocrine disease	11 (29.7)	9 (10.2)	0.007
**Clinical data**			
Acute MI	16 (42.1)	43 (47.3)	0.593
Chronic coronary syndrome	17 (44.7)	44 (48.4)	0.708
Arythmia	15 (39.5)	41 (45.1)	0.560
Heart failure	17 (44.7)	50 (59.9)	0.290

n: Frequency; %: Percentage; *Pearson’s Chi-squared test.

Smoking was the most frequent cardiovascular risk factor, with 66.6% of patients in both groups being active smokers, 53.4% had hypertension (50% in the intervention group and 59% in the control group), 45.7% were diabetic (44.7% in the intervention group and 46.2% in the control group), 20 patients were monitored for an endocrine pathology (29.7% in the intervention group and 10.2% in the control group) including thyroid disorders (hypothyroidism or hyperthyroidism), and other metabolic endocrine conditions such as adrenal or pituitary disorders.

Furthermore, the two groups were comparable in terms of major cardiac conditions and complications at baseline.

### Biological parameters

At baseline (Table 2), the intervention and control groups were generally comparable across most biochemical parameters. Total cholesterol was slightly lower in the intervention group, and LDL and HbA1c levels were similar between groups, indicating minimal differences. Triglycerides were notably higher and HDL lower in the intervention group, reflecting large and moderate baseline differences, respectively.

**Table 2:** Biological parameters of the study population.

Biological parameters	Group		P-value*	Cohen’s d
	Intervention (N = 38) M ± (SD)	Control (N = 91) M ± (SD)		
Total cholesterol**(mmol/L)**	4.32 (1.47)	4.9 (2.41)	0.164	−0.29
Triglycerides**(mmol/L)**	2.14 (0.88)	1.3 (0.54)	**<0.001**	**1.15**
LDL**(mmol/L)**	2.5 (1.45)	2.5 (1.55)	0.425	0
HDL**(mmol/L)**	1.05 (0.7)	1.5 (1.45)	**0.004**	**−0.40**
Glycated hemoglobin (%)	8 (2)	7.8 (2)	0.602	0.10

M: Mean;SD: Standard Deviation; * Student’s t-test for independent samples.

### Initial assessment of knowledge

Table 3 presents the baseline assessment of knowledge in the intervention and control groups. At baseline, participants in the intervention group demonstrated significantly higher knowledge than those in the control group in several domains. In particular, 71.1% of participants in the intervention group correctly identified cardiovascular risk factors compared with 44.0% in the control group (*p* = 0.005). Regarding lifelong aspirin therapy after myocardial infarction, the proportions of correct responses were similar between the intervention and control groups (39.5% vs. 42.9%, *p* = 0.723). In contrast, knowledge regarding statin treatment was significantly higher in the intervention group than in the control group (52.6% vs. 30.8%, *p* = 0.019).

**Table 3:** Initial assessment of knowledge of the study population.

Assessed criterion (correct answer)	Intervention group n (%)	Control group n (%)	P-value*
Cause of coronary heart disease	17 (44.7)	32 (35.2)	0.307
Cardiovascular risk factors	27 (71.1)	40 (44)	**0.005**
Representation of coronary heart disease	19 (51.4)	35 (38.5)	0.181
Characteristics of chest pain	35 (92.1)	49 (53.8)	**<0.001**
Screening Examinations	27 (71.1)	44 (48.9)	**0.021**
Coronary angiography is essential	33 (86.8)	53 (58.2)	**0.002**
Radial approach of coronary angiography	29 (76.3)	46 (51.1)	**0.008**
Femoral route of coronary angiography	24 (63.2)	42 (46.2)	0.078
General Anesthesia for Coronary Angiography	4 (10.5)	22 (24.2)	0.078
Complication of coronary angiography	6 (15.8)	26 (28.6)	0.125
Percutaneous revascularization	9 (23.7)	19 (20.9)	0.725
Types of stents	4 (10.5)	21 (23.1)	0.100
Risk of restenosis	23 (60.5)	41 (45.1)	0.109
Duration of aspirin therapy	15 (39.5)	39 (42.9)	0.723
Duration of treatment with clopidogrel	8 (21.1)	18 (19.8)	0.870
Duration of treatment with Statin	20 (52.6)	28 (30.8)	**0.019**
Risk Factor Control	32 (86.5)	39 (43.3)	**<0.001**
Blood Pressure Objectives	6 (15.8)	13 (14.3)	0.826
Objectives of glycated hemoglobin	3 (7.9)	10 (11)	0.595
Objectives of Low-density lipoproteins cholesterol	0	6 (6.6)	0.179
Smoking cessation is mandatory	32 (84.2)	53 (58.2)	**0.005**
Emergency assistance number	10 (26.3)	18 (19.8)	0.412
Physical activity after MI	16 (42.1)	37 (40.7)	0.879
Fish consumption	10 (26.3)	19 (20.9)	0.500
Fruit and vegetable consumption	28 (73.7)	59 (64.8)	0.328
We must reduce the consumption of red meat	33 (86.8)	61 (67)	0.021
Olive oil is better than vegetable oil	14 (36.8)	29 (31.9)	0.585
Salt excess is harmful	36 (94.7)	59 (64.8)	**<0.001**

n: Frequency; %: Percentage; * Pearson’s Chi-squared.

### Evaluation of the TPE program

#### Evaluation of overall program satisfaction.

Overall satisfaction with the therapeutic education program was high. All participants reported an overall satisfaction score ≥8/10 on a 0–10 scale. Participants particularly appreciated the organization of the therapeutic sessions, the quality of information provided, and the educational materials distributed. They reported that their questions were addressed and that the program met their expectations. However, some participants considered the session schedule unsuitable.

#### Immediate and medium-term knowledge assessment in the intervention group.

The assessment of knowledge revealed a notable enhancement in the majority of the aspects assessed immediately following the completion of the educational program, about the illness, its diagnostic procedures, the therapies, and the sanitary and nutritional guidelines to adhere to, displaying a significant variance in statistical terms. This enhancement remains statistically significant at the 3-month assessment; nevertheless, it was statistically significant only for the objectives related to low-density lipoproteins cholesterol and the complications associated with coronary angiography (Table 4).

**Table 4:** Immediate and medium-term assessment of the knowledge of patients in the intervention group.

Assessed criterion (correct answer)	Initial assessment (T0) n (%)	Just after intervention (T1) n (%)	After 3 months (T2) n (%)	p-value^1^	p-value (T1/T0)^2^	p-value (T2/T0)^2^	p-value (T2/T1) ^2^
Cause of coronary heart disease	17 (44.7)	29 (76.3)	23 (60.5)	**<0.001**	**<0.001**	**<0.001**	1
Cardiovascular risk factors	27 (71.1)	30 (78.9)	23 (60.5)	0.102	**0.039**	0.219	1
Representation of coronary heart disease	19 (51.4)	30 (78.9)	21 (55.3)	**0.002**	**0.001**	**0.021**	0.625
Characteristics of chest pain	35 (92.1)	29 (76.3)	23 (60.5)	0.779	1	1	1
Screening Examinations	27 (71.1)	25 (65.8)	21 (55.3)	0.156	0.063	0.453	1
Coronary angiography is essential	33 (86.8)	28 (73.7)	24 (63.2)	0.368	0.625	–	1
Radial approach of coronary angiography	29 (76.3)	31 (81.6)	22 (57.9)	0.091	**0.016**	0.625	0.500
Femoral route of coronary angiography	24 (63.2)	31 (81.6)	22 (57.9)	**0.030**	**0.001**	0.289	0.500
General Anesthesia for Coronary Angiography	4 (10.5)	1(1.63)	4 (10.5)	0.311	0.500	1	0.375
Complication of coronary angiography	6 (15.8)	30 (78.9)	14 (36.8)	**<0.001**	**<0.001**	**0.012**	**0.004**
Percutaneous revascularization	9 (23.7)	2 (5.3)	8 (21.1)	0.105	0.289	0.453	0.109
Types of stents	4 (10.5)	29 (76.3)	20 (52.6)	**<0.001**	**<0.001**	**<0.001**	0.625
Risk of restenosis	23 (60.5)	29 (76.3)	23 (60.5)	**<0.001**	**0.021**	**0.008**	1
Duration of aspirin therapy	15 (39.5)	31 (81.6)	22 (57.9)	**<0.001**	**<0.001**	**0.004**	0.500
Duration of treatment with clopidogrel	8 (21.1)	31 (81.6)	19 (50)	**<0.001**	**<0.001**	**0.002**	0.063
Duration of treatment with Statin	20 (52.6)	30 (78.9)	24 (63.2)	**<0.001**	**0.001**	–	1
Risk Factor Control	32 (86.5)	30 (78.9)	24 (63.2)	0.135	0.625	–	1
Blood Pressure Objectives	6 (15.8)	25 (65.8)	15 (39.5)	**<0.001**	**<0.001**	**0.012**	0.146
Targets of glycated hemoglobin	3 (7.9)	21 (55.3)	15 (39.5)	**<0.001**	**<0.001**	**<0.001**	0.754
Targets of Low-density lipoproteins cholesterol	0 (0)	19 (50)	5 (13.2)	**<0.001**	**<0.001**	**–**	**0.006**
Smoking cessation is mandatory	32 (84.2)	29 (76.3)	24 (63.2)	0.368	1	–	0.500
Emergency assistance number	10 (26.3)	31 (81.6)	19 (50)	**<0.001**	**<0.001**	**<0.001**	0.125
Physical activity after MI	16 (42.1)	6 (15.8)	7 (18.4)	0.115	0.118	0.227	0.774
Fish consumption	10 (26.3)	3 (7.9)	0 (0)	**0.016**	0.180	–	0.250
Fruit and vegetable consumption	28 (73.3)	31 (81.6)	21 (55.3)	0.066	**0.004**	0.452	0.500
We must reduce the consumption of red meat	33 (86.8)	30 (78.9)	21 (55.3)	0.607	0.375	1	1
Olive oil is better than vegetable oil	14 (36.8)	2 (5.3)	7 (18.4)	0.103	**0.039**	1	0.180
Salt excess is harmful	36 (94.7)	31 (81.6)	23 (60.5)	0.135	0.500	–	1

n: Frequency; %: Percentage; ^**1**^ Cochrane Q test; ^**2**^ Mc Nemar’s test.

#### Assessment of biological parameters.

At 3 months, significant changes in glycated hemoglobin and lipid profile parameters were observed among participants in the intervention group compared with baseline measurements. An increase in the proportion of patients achieving LDL targets was also observed (Table 5).

**Table 5:** Biological parameters evaluation of the study population.

Biological Parameter	Baseline assessment Mean ± (standard deviation)		Cohen’s d	Mean difference (95% CI)	p-value^1^	At 3 months Mean ± (standard deviation)		Cohen’s d	Mean difference (95% CI)	p-value^1^	p-value (baseline vs at 3 months in intervention group)^2^
	Intervention Group	Control Group				Intervention Group	Control Group				
**Total Cholesterol** **(mmol/L)**	4.32 ± 1.47	4.9 ± 2.41	−0.29	0.59 (−0.24 to 1.42)	0.164	3.75 ± 1.56	5.08 ± 2.41	**−0.66**	**1.33** **(0.49to2.17)**	**0.002**	**0.002**
**Triglycerides** **(mmol/L)**	2.14 ± 0.88	1.30 ± 0.54	**1.15**	**−0.84** **(−1.15to-0.53)**	**<0.001**	1.70 ± 0.78	1.30 ± 0.53	**0.60**	**−0.37** **(−0.65to-0.10)**	**0.010**	**<0.001**
**LDL** **(mmol/L)**	2.5 ± 1.45	2.50 ± 1.55	0	0.24 (−0.35to0.82)	0.425	1.88 ± 1.11	2.90 ± 1.60	**−0.74**	**1.02** **(0.45to1.59)**	**<0.001**	**<0.001**
**HDL** **(mmol/L)**	1.05 ± 0.70	1.50 ± 1.45	**−0.40**	**0.46** **(0.15to0.77)**	**0.004**	1.23 ± 0.72	1.50 ± 1.09	−0.29	0.30 (−0.03to0.62)	0.074	**0.005**
**Glycated hemoglobin (%)**	8.1 ± 2	7.8 ± 2	0.10	−0.21 (−0.98to0.57)	0.602	7.50 ± 1.81	8.00 ± 2.18	−0.25	0.50 (−0.29to1.30)	0.212	**0.001**

^**1**^ Student’s t-test for independent sample; ^**2**^ Paired Student’s t-test.

A comparison of these same parameters 3 months after the TPE program between the intervention and control groups showed that patients in the intervention group had significantly lower levels of total cholesterol, triglycerides, and LDL than patients in the control group (Table 5). In fact, at follow-up, the intervention and control groups showed varying differences across the biochemical parameters. Total cholesterol and LDL were lower in the intervention group compared with the control group, with moderate-to-large effect sizes (Cohen’s d = −0.66 and −0.74, respectively). Triglycerides were higher in the intervention group, with a moderate effect size (Cohen’s d = 0.60). HDL and glycated hemoglobin (HbA1c) showed smaller differences between groups, with small effect sizes (Cohen’s d = −0.29 and −0.25, respectively). Overall, these effect sizes suggest that the intervention had the greatest impact on total cholesterol, triglycerides, and LDL, while the effects on HDL and HbA1c were more modest.

Furthermore, to account for baseline differences between groups, we calculated the change (Δ) from baseline to 3 months for all biological parameters. The intervention group showed significant improvements compared to the control group. Total cholesterol decreased by −0.57 ± 0.80 mmol/L in the intervention group, whereas it increased by +0.18 ± 0.60 mmol/L in the control group (p = 0.002). LDL cholesterol decreased by −0.62 ± 0.70 mmol/L in the intervention group, compared to an increase of +0.40 ± 0.85 mmol/L in the control group (p < 0.001). Triglycerides decreased by −0.44 ± 0.50 mmol/L in the intervention group versus no change in the control group (0.00 ± 0.45 mmol/L, p < 0.001). HDL increased by +0.18 ± 0.25 mmol/L in the intervention group and remained unchanged in the control group (0.00 ± 0.20 mmol/L, p = 0.005). Glycated hemoglobin decreased by −0.60 ± 0.80% in the intervention group, compared to an increase of +0.20 ± 0.85% in the control group (p < 0.001).

These results demonstrate that the TPE program significantly improved lipid profiles and glycemic control in patients with CHD, independent of baseline differences.

#### Evaluation of QoL using the SF12 questionnaire.

At 3 months, patients in the intervention group demonstrated significantly higher SF-12 scores in several domains compared with the control group (Table 6). Improvements were particularly evident in physical role (median [IQR]: 8 [5–8] vs. 6 [4–8]; p = 0.019), emotional role (8 [7–8] vs. 7 [5–8]; p = 0.002), body pain (3 [3–4] vs. 2 [2–4]; p = 0.001), and social functioning (4 [4–4] vs. 4 [4–3]; p = 0.028). Additionally, both the physical health summary score (18 [15–20] vs. 15 [11–20]; p = 0.025) and mental health summary score (21 [18.75–23] vs. 20 [16.75–23]; p = 0.024) were significantly higher in the intervention group.

**Table 6:** Evaluation of QoL using the SF12 questionnaire.

SF12 elements (median [IQR])	Group		P-value*
	Intervention (n = 38)	Control (n = 91)	
General Health	3 [2–3]	3 [2–3]	0.079
Physical functioning	4 [4–5]	4 [2 –5]	0.381
Physical role	8 [5 –8]	6 [4 –8]	**0.019**
Emotional role	8 [7–8]	7 [5 –8]	**0.002**
Body pain	3 [3–4]	2 [2 –4]	**0.001**
Mental health	7 [6 –8]	6,5 [5 –8]	0.337
Vitality	3 [2–3]	3 [2–3]	0.236
Social functioning	4 [4–4]	4 [4–3]	**0.028**
Physical health score	18 [15 –20]	15 [11 –20]	**0.025**
Mental health score	21 [18.75–23]	20 [16.75–23]	**0.024**

IQR: Interquartile Range; * Mann-Whitney U test.

#### Long-term assessment: Occurrence of cardiovascular events at 1 year.

At one year post-intervention, the occurrence of at least one cardiovascular event differed between groups, with fewer patients in the intervention group experiencing a composite cardiovascular outcome compared with the control group. Specifically, 8 out of 38 patients (21.1%; 95% CI: 9.4%–38.6%) in the intervention group experienced at least one cardiovascular event, compared with 54 of 91 patients (59.3%; 95% CI: 48.0%–70.0%) in the control group (p < 0.001). This corresponds to a relative risk (RR) of 0.36 (95% CI: 0.18–0.72).

The individual components of the composite cardiovascular outcome showed different patterns between groups. Acute myocardial infarction occurred only in the control group (5.6%), while coronary revascularization was reported only among patients in the intervention group (25%). Hospitalizations for heart failure were more frequent in the control group compared with the intervention group (44.4% vs. 25%). Stable angina was reported with similar frequency in both groups (50%). These findings highlight the heterogeneity of the composite outcome components. While the higher overall event rate in the control group was mainly driven by clinically severe outcomes, including myocardial infarction and heart failure hospitalization, the occurrence of revascularization in the intervention group may reflect planned secondary prevention strategies and clinical management decisions rather than disease progression alone. Therefore, individual cardiovascular outcomes should be interpreted separately in addition to the composite endpoint.

## Discussion

Improving the management of coronary patients requires a proactive, personalized approach based primarily on Therapeutic Patient Education (TPE), effectively integrating individual, cultural, and technological dimensions to reinforce adherence and optimize clinical outcomes.

The importance of structured therapeutic patient education (TPE) as an integral component of coronary disease management is reiterated in contemporary clinical guidelines, which emphasize continuity of education across the inpatient–outpatient transition to support long-term self-management. The 2021 European Society of Cardiology (ESC) Guidelines on the prevention of cardiovascular disease emphasize lifestyle modification and patient education as key components of secondary prevention and suggest coordinated, continuous educational interventions as part of standard care [19]. The 2020 ESC guidelines for the treatment of acute coronary syndromes without persistent ST-segment elevation also emphasize the necessity of providing comprehensive secondary-prevention strategies throughout the patient’s care pathway, such as counseling, risk-factor modification, and structured rehabilitation/education [20]. Education, psychosocial risk-factor treatment, and the integration of inpatient initiation with outpatient follow-up are further supported as class-level priority for patients at high cardiovascular risk by major North American statements on cardiac rehabilitation and core program components [21].

Before the educational program began, baseline knowledge varied between the intervention and control groups. Although several domains were comparable, participants in the intervention group demonstrated significantly higher knowledge for certain aspects of coronary heart disease, including cardiovascular risk factors, selected diagnostic procedures, treatment, and lifestyle recommendations. Nevertheless, both groups exhibited important knowledge gaps, particularly regarding pain characteristics and the management of cardiovascular risk factors. These findings are consistent with a recent multicenter randomized trial conducted by Li et al. (2022) [22], which included 608 myocardial infarction survivors and compared a narrative psychoeducational intervention with standard didactic education. The study reported that baseline knowledge levels were similar between groups, and although the intervention improved attitudes and care-seeking beliefs, knowledge scores related to myocardial infarction symptoms did not significantly differ between the two groups at follow-up [22].

A quasi-experimental design was selected to evaluate the TPE program in conditions closely aligned with routine clinical practice, thereby strengthening the external validity and real-world relevance of the findings. In our setting, patient preferences played a substantial role: some individuals expressed strong interest in participating in the TPE program, whereas others were hesitant due to personal, cultural, or logistical constraints. The quasi-experimental approach allowed us to account for these preferences and to allocate participants accordingly, resulting in an intervention group composed of individuals who were motivated and more likely to engage fully with the program. This design choice thus reflects the pragmatic realities of clinical implementation and enhances the applicability of our results to everyday practice.

The results of our post-TPE study demonstrated significant improvements in patients’ knowledge following the intervention. Although TPE programs are designed to promote healthier behaviors and treatment adherence, these outcomes were not directly assessed in our study and may represent potential explanations for the observed findings. Similar effects have been documented in more recent literature, where educational interventions significantly impact health-related knowledge and behaviors. For instance, a randomized controlled trial of planned discharge education in 100 acute myocardial infarction (AMI) patients found that a structured education program markedly increased patients’ knowledge of cardiovascular risk factors, beliefs about medication and diet, as well as adherence to follow-up after four weeks [23]. Moreover, a quasi-experimental study by Arman et al. (2025), based on the Theory of Planned Behavior, showed that a ten-session family-supported education intervention among 140 post-MI patients significantly improved self-care behaviors, behavioral intentions, and mobilized more favorable attitudes and perceived behavioral control at 4-month follow-up [24]. These findings reinforce the pivotal role of TPE in enhancing long-term adherence and self-management in coronary patients.

Comparative analysis between the post-education TPE group and the control group regarding lipid balance revealed that educated patients had significantly lower mean levels of total cholesterol (3.75 vs. 5.08; p = 0.002), triglycerides (1.7 vs. 1.3; p = 0.010) and LDL (1.8 vs. 2.9; p = 0.01). Patients in the intervention group also had a higher LDL at objectives (<0.55g/l) than patients in the control group, with a statistically significant difference (p < 0.001). In addition, we noted a clear improvement in the control lipid profile at 3 months in the “Educated” group, with a significant difference in the entire lipid profile, with mean levels of total cholesterol (3. 75 vs. 4.32; p = 0.002), triglycerides (1.7 vs. 2.14; p < 0.001) and LDL (1.88 vs. 2.53; p < 0.001), as well as mean glycated hemoglobin (7.5 vs. 8.1; p = 0.001).

The magnitude of these differences, quantified by Cohen’s d, highlights the clinical relevance of the intervention. For example, the effect size for total cholesterol (d = −0.66) and LDL (d = −0.74) indicates a moderate-to-large reduction, suggesting that the TPE program had a substantial impact on these key cardiovascular risk factors. Triglycerides showed a positive effect size (d = 0.60), reflecting a moderate improvement, while changes in HDL and HbA1c were smaller (d = −0.29 and −0.25, respectively), indicating modest effects. These effect sizes complement the p-values by demonstrating that the intervention not only produced statistically significant differences but also clinically meaningful improvements in lipid and glycemic control.

Although the intervention group demonstrated significant improvements in lipid profile and glycated hemoglobin, these changes should be interpreted with caution. Medication adherence, statin intensity, modifications in lipid-lowering therapy, antidiabetic treatment adjustments, and other cardiovascular medication changes during follow-up were not systematically recorded. Consequently, the observed biological improvements may have resulted from both pharmacological management and participation in the therapeutic patient education program, and the independent contribution of the educational intervention cannot be determined.

These results are consistent with more current data. In fact, it has been demonstrated that a patient-centered teaching strategy considerably lowers cholesterol levels in individuals with dyslipidemia [25].

Furthermore, very high-risk individuals with atherosclerotic cardiovascular disease continue to lack understanding of LDL-C treatment targets, underscoring the continuous need for systematic educational interventions in this population [26]. The relevance of lifestyle education, which includes dietary modifications, physical exercise, and self-management, in lowering cardiovascular risk is further supported by a more comprehensive synthesis of secondary prevention measures [27].

QoL was assessed using the SF-12 questionnaire, which evaluates physical, emotional, and social domains of health status. At the 3-month assessment, participants in the TPE group reported higher SF-12 scores than the control group, particularly in physical role, emotional role, and social functioning. Higher scores indicate better perceived health, suggesting that patients exposed to the TPE program may experience improved functional and psychological well-being. While these differences are consistent with previous studies reporting potential benefits of educational and cardiac rehabilitation programs on quality of life (QoL) [28–31], the absence of baseline QoL measurements in our study limits the interpretation of these findings. Therefore, the observed differences at three months should be considered as cross-sectional associations between group participation and QoL domains rather than evidence of QoL improvement attributable to the TPE intervention. Considering the published minimal important differences (MIDs) for SF-12 components, the observed differences may suggest potential clinical relevance; however, these findings should be interpreted cautiously, as pre-existing differences between groups cannot be excluded. Future studies incorporating baseline and longitudinal QoL assessments are needed to evaluate changes over time and better determine the potential impact of TPE programs on patients’ quality of life.

At the one-year follow-up, the occurrence of cardiovascular events was lower among patients who received therapeutic education compared with those who did not receive the intervention. Specifically, cardiovascular events occurred in 21.1% of patients in the intervention group compared with 59.3% in the control group (p < 0.001). These findings suggest an association between participation in the therapeutic education program and a lower incidence of cardiovascular events over one year. This association may be explained by several mechanisms, including improved disease-related knowledge, enhanced self-management skills, better adherence to recommended treatments, and adoption of healthier behaviors. However, given the quasi-experimental, non-randomized design of the study, these findings should be interpreted cautiously, as residual confounding and baseline differences between groups cannot be completely excluded.

The observed one-year incidence of cardiovascular events in the control group (59.3%) appears higher than rates reported in several international studies. For example, large registries such as the REACH registry have reported one-year event rates of approximately 12–14% for composite outcomes including cardiovascular death, myocardial infarction, stroke, or hospitalization [32]. Similarly, studies conducted among post-myocardial infarction populations have reported lower rates of rehospitalization for acute coronary syndromes or revascularization, generally ranging from 6% to 12% within one year [33]. However, higher event rates have been described in real-world cohorts of high-risk patients. Studies involving individuals with established cardiovascular disease and multiple comorbidities have reported major adverse cardiovascular event rates ranging from 20 to 40 events per 100 person-years [34]. Furthermore, recurrent cardiovascular events are known to occur frequently during the first year following an acute coronary event, particularly among vulnerable populations with multiple risk factors [35].

In our study, the elevated event rate observed in the control group may be related to the high-risk clinical profile of the study population, including a high prevalence of smoking, hypertension, diabetes, and suboptimal lipid control at baseline. In addition, differences in the definition of cardiovascular outcomes across studies may explain variations in reported event rates. The composite outcome used in the present study included a broad range of cardiovascular events, including hospitalizations and revascularization procedures, which may have contributed to the higher overall event rate compared with studies using more restrictive endpoints.

Analysis of individual cardiovascular outcomes revealed different patterns between groups. Acute myocardial infarction occurred only in the control group (5.6%), whereas coronary revascularization was observed exclusively among patients in the intervention group (25%). Hospitalizations for heart failure were more frequent among patients in the control group compared with the intervention group (44.4% vs. 25%), while stable angina was reported at similar rates in both groups (50%). These findings highlight the heterogeneity of the composite cardiovascular outcome, as its individual components differ in terms of clinical severity, prognostic significance, and underlying mechanisms. In particular, coronary revascularization may reflect not only disease progression but also clinical decision-making, patient management strategies, and healthcare access. Therefore, the observed differences in the composite outcome should be interpreted alongside the individual event components rather than considered as a uniform measure of cardiovascular risk.

Overall, the lower incidence of cardiovascular events observed in the intervention group may reflect multiple factors, including improved adherence to recommended therapies, better risk-factor management, enhanced recognition of warning symptoms, and earlier healthcare-seeking behaviors following participation in the therapeutic education program. Nevertheless, these findings cannot be interpreted as definitive evidence of a causal effect of the intervention, as the non-randomized design, baseline imbalances, and absence of adjusted analyses limit the ability to isolate the independent contribution of therapeutic education to clinical outcomes.

Additionally, the exact timing of cardiovascular events was not collected, precluding time-to-event analyses. Therefore, survival analyses such as Kaplan-Meier curves and Cox regression models could not be performed. Future studies should incorporate prospective collection of event timing to allow a more comprehensive evaluation of cardiovascular risk over time.

Nevertheless, the consistency of improvements observed across knowledge, adherence, and biological indicators supports the plausibility of a beneficial association between therapeutic education and patient outcomes.

Although recent randomized controlled trials of standard cardiac rehabilitation (CR) programs frequently do not show large effects on composite primary endpoints (such as cardiovascular death, non-fatal MI, or rehospitalization), more recent meta-analyses do support beneficial effects of CR. For instance, a contemporary meta-analysis of 85 RCTs demonstrated that exercise-based CR is associated with significant reductions in cardiovascular mortality (RR = 0.74), recurrent myocardial infarction (RR = 0.82), and hospitalizations, compared to usual care [36]. Moreover, a randomized controlled trial published in 2021 showed that a long-term hospital-based secondary prevention program significantly lowered recurrent events in coronary heart disease patients [37].

These findings underscore that structured education and rehabilitation interventions can effectively reduce major adverse cardiovascular events in real-world populations.

The observed 59.3% one-year incidence of major cardiovascular events in our control group, although higher than some international benchmarks, likely reflects the exceptionally high-risk profile of our study population and the specificities of the Tunisian healthcare system. At baseline, non-educated patients had a very high prevalence of active smoking (66.6%), hypertension (54.9%), and diabetes (46.2%), with only 30.8% achieving LDL targets, putting them at elevated cardiovascular risk.

Contributing contextual factors include poor long-term medication adherence, constrained access to specialist care, and socio-economic barriers to lifestyle modification, challenges well documented in Tunisia. For instance, a national hypertension registry (NaTuRe HTN) reported suboptimal blood pressure control, especially among high-risk patients in the public sector [38].In parallel, a study evaluating LDL-C target achievement after acute coronary syndrome in Tunisia found that less than one-third of patients reached European guideline goals (LDL-C < 0.55 g/L), underscoring persistent gaps in lipid management [39].

The findings of this study suggest that TPE can be successfully scaled and implemented in a variety of healthcare settings, including hospitals, outpatient clinics, and community health centers. At a minimum, effective implementation requires trained healthcare personnel (such as nurses, educators, or allied health professionals), structured educational sessions, standardized teaching materials, and suitable facilities for individual or group interactions. Among the four stages of the program, patient assessment and individualized education planning, practical skills training for self-management, and reinforcement sessions appear to be most critical for achieving clinically meaningful outcomes, whereas less resource-intensive components, such as distribution of educational leaflets, provide supportive but not essential benefits. The program’s success also depended on cultural and linguistic tailoring, with materials delivered in Arabic and adapted to local health beliefs and practices, highlighting the importance of context-specific education in patient engagement and adherence.

Although a formal cost-effectiveness analysis was not conducted, the intervention primarily involves personnel time and educational materials rather than expensive technology or equipment, suggesting that it may be feasible even in resource-limited settings. For broader adoption, healthcare systems should prioritize staff training, ensure regular follow-up, and adapt materials to the local language and cultural context. In addition, sustainability of benefits over time is a key consideration: knowledge retention declined between immediate post-intervention and 3 months, and long-term compliance with lifestyle modifications remains challenging. Strategies such as booster sessions, digital follow-up platforms, and organized medical and paramedical networks for ongoing TPE could help maintain knowledge, reinforce behavior change, and sustain improvements in biological and clinical outcomes. Future research should evaluate the long-term effectiveness of these strategies, including their cost-effectiveness, adaptability across healthcare systems, and impact on patient-centered outcomes.

### Limitations

This study has several limitations. First, although randomized controlled trials represent the gold standard for evaluating interventions, a quasi-experimental design was adopted because of logistical constraints in a real-world Tunisian university hospital. Consequently, the absence of random allocation may have resulted in systematic differences between groups. Baseline imbalances, including differences in endocrine disorders, lipid parameters, educational level, and baseline knowledge, may have introduced residual confounding and influenced the observed outcomes. Although efforts were made to account for these differences through appropriate comparisons, the possibility that unmeasured or incompletely measured factors contributed to the observed associations cannot be excluded. Given the limited sample size, multivariable or propensity score analyses were not performed because they could have produced unstable estimates and potentially misleading adjustments.

Second, participation in the intervention required patients’ consent, which may have introduced self-selection bias. Patients who agreed to participate may have been more motivated, more engaged in self-management behaviors, or more receptive to lifestyle recommendations than those who declined participation, potentially contributing to the observed improvements. Furthermore, recruitment from the same department during the same period may have allowed contamination through informal information exchange between participants and non-participants. Therefore, the magnitude of the observed effects should be interpreted cautiously, and the findings should not be considered evidence of causality.

Third, medication adherence and treatment modifications during follow-up, including statin intensity and antidiabetic therapy, were not systematically recorded and may have contributed to the observed changes in lipid profile and glycated hemoglobin. Additional limitations include the inability to perform subgroup or time-to-event analyses because of the limited sample size and the absence of event timing data, the use of a composite cardiovascular endpoint comprising outcomes of varying clinical importance, the lack of baseline quality-of-life assessment, the absence of formal psychometric validation of the knowledge questionnaire, and the lack of blinding of outcome assessors. Moreover, the timing of follow-up initiation differed between groups: in the intervention group, the one-year follow-up period for cardiovascular outcomes was initiated after completion of the TPE program, whereas in the control group, follow-up started after hospital discharge. Although this approach was consistent with the intervention design, this difference in the observation period relative to discharge may have influenced the comparability of event rates between groups and should be considered when interpreting the findings. Furthermore, longitudinal changes in knowledge were assessed only in the intervention group, while knowledge was compared between groups at baseline. Therefore, although the observed improvement in knowledge following the TPE program suggests a beneficial effect, the contribution of other factors, such as the passage of time, routine medical care, or patients’ independent access to health information, cannot be completely excluded.

Furthermore, satisfaction with the therapeutic education program was assessed using self-reported measures collected at the end of the intervention. Although satisfaction scores were high, detailed response distributions were not available for further analysis, and these findings may have been influenced by social desirability bias or participants’ positive perception of receiving an educational intervention. Therefore, satisfaction results should be interpreted primarily as indicators of program acceptability rather than evidence of intervention effectiveness.

Finally, the single-center design may limit the generalizability of our findings, although the pragmatic nature of the study enhances their relevance to similar resource-limited settings. Future multicenter randomized studies with larger sample sizes, validated assessment tools, comprehensive medication monitoring, blinded outcome assessment, repeated knowledge assessments in both groups, and appropriate statistical adjustment are warranted to confirm these findings.

## Conclusion

Therapeutic patient education (TPE) is an important component of chronic disease management, supporting patient autonomy and self-management. In this quasi-experimental study, participation in a TPE program was associated with higher levels of knowledge regarding coronary heart disease, cardiovascular risk factors, screening, and treatments. The intervention group also showed favorable changes in some biological parameters, including lipid profiles and glycated hemoglobin. Furthermore, a lower incidence of cardiovascular events was observed in the intervention group during the one-year follow-up period. However, these findings should be interpreted cautiously, as the study design does not allow definitive conclusions regarding the causal effectiveness of TPE, and residual confounding and selection bias may have influenced the observed associations.

These results support the potential value of integrating TPE into routine cardiovascular care to promote patient-centered, proactive, and personalized management, particularly in resource-limited settings. Future research using randomized controlled designs and larger multicenter samples is needed to confirm these associations, strengthen causal inference, and improve generalizability. Future studies should also incorporate appropriate adjustment strategies, including multivariable analyses or propensity score approaches, to better account for baseline differences and potential confounding factors. In addition, longitudinal assessment of quality of life, patient engagement, long-term adherence, and sustainability of TPE programs will be important to further characterize their contribution to chronic cardiovascular disease management.

## Supporting information

S1 Dataset Raw anonymized data used for the analysis. (XLSX)

S1 File Ethical Approval. (PDF)

S2 File PLOSOne Human Subjects Research Checklist. (DOCX)

S3 File Protocole in French. (DOCX)

S4 File Translation of the Protocole. (DOCX)

S5 File Trends checklist. (DOC)
