# Supplementary material for: Evaluation of a therapeutic education program for patients with coronary heart disease in a Tunisian university hospital: A quasi-experimental study
Source: PLoS One. 2026 Nov 9;21(11):e0361814. doi: 10.1371/journal.pone.0361814 (PMC13747245; doi:10.1371/journal.pone.0361814)
Supplement: Supplementary file 1 [file pone.0361814.s002.PDF]

Ethical approval

The members of the Ethics Committee of CHU Sahloul Sousse, meeting on April 20, 2022, attest that the research project entitled "Education thérapeutique du patient coronarien : Création d'une plateforme digitale de suivi", proposed by Dr Hela Ghali et al.

is registered under number number HS 12-2022.

The ethics committee grants Dr Hela Ghali authorization to carry out this research project while respecting the methodology presented in the application for project approval.

Professor Ajmi Chaouech

Chair of the Ethics and Research Committee

HOPITAL SAHLOUL DE SOUSSE
Pr Ajmi Chaouech
Professeur
en Anesthésie - Réanimation

Hopital Sahloul de Sousse
Pr. Ajmi CHAOUCH
Président du Comité d'Ethique
