# Supplementary material for: Evaluation of a therapeutic education program for patients with coronary heart disease in a Tunisian university hospital: A quasi-experimental study
Source: PLoS One. 2026 Nov 9;21(11):e0361814. doi: 10.1371/journal.pone.0361814 (PMC13747245; doi:10.1371/journal.pone.0361814)
Supplement: Supplementary file 3 [file pone.0361814.s004.docx]

**Education thérapeutique du patient coronarien : création d’une Plateforme digitale de télé-suivi**

**Dr Hela Ghali**

Introduction

Les maladies cardiovasculaires (MCV) constituent un problème de santé mondial important (1,2). Selon l'Organisation mondiale de la santé (OMS), les MCV sont responsables de 17,5 millions de décès en 2012 et plus des trois quarts des décès de MCV ont eu lieu dans des pays à revenu faible ou intermédiaire (3).

Selon un récent rapport publié par l'OMS, les maladies cardiovasculaires constituent la principale cause de décès en Tunisie loin devant les maladies infectieuses (4).

Plus fort que le VIH, les cancers ou encore le diabète, les maladies cardiovasculaires, constituent la première cause de la mortalité en Tunisie avec 49% de la totalité des décès.

Devant l’augmentation du nombre de maladies chroniques avec le vieillissement de la population, et devant la constatation de l’insuffisance du contrôle thérapeutique, un nouveau besoin s’est émergé, celui d’avoir un patient partenaire de soins.

L’éducation thérapeutique est une approche humaniste centrée sur le patient, ses besoins et ses ressources. Elle est proposée non seulement pour aider le patient à comprendre sa maladie et son traitement mais aussi pour l’aider à devenir autonome.

La prise en charge des facteurs de risque modifiables reste une priorité, tant en prévention primaire que secondaire, afin d’améliorer le pronostic de cette affection redoutable dont le caractère asymptomatique n’est pas synonyme de bénignité. L’aide au sevrage du tabac, une activité physique régulière, une alimentation cardio-protectrice (régime « méditerranéen »), la gestion du stress, une bonne adhésion au traitement médicamenteux qui améliore son observance, un recours judicieux au système de santé, l’aide à la réinsertion professionnelle sont les enjeux actuels de l’éducation thérapeutique (ETP) du patient coronarien (5).

L’intérêt de l’ETP du patient coronarien a été mis en évidence par plusieurs types d’approches.

Cependant, dans la plupart des programmes d'éducation thérapeutique, le suivi du patient s'arrête à la fin du programme d'éducation. Or, il est essentiel d'accompagner le patient coronarien dans la troisième phase de sa prise en charge. Ainsi, notre étude vise à créer une plateforme numérique permettant le suivi des patients après avoir bénéficié d'un programme d'éducation thérapeutique.

Dans ce cadre, nous avons choisi de planifier un programme d’éducation thérapeutique chez les patients coronariens consultants le service de cardiologie de l’hôpital universitaire Sahloul, en collaboration avec le service de Prévention et Sécurité des soins.

Le but des séances de l’ETP est de conférer aux patients coronariens les moyens pour maintenir les compétences à fin d’améliorer leur état de santé, leur qualité de vie et les responsabiliser quant à la prise en charge de leur maladie, afin de mieux vivre avec leur maladie. Ainsi, les objectifs seront :

- Améliorer l’observance thérapeutique du patient
- Adopter un comportement alimentaire sain
- Aider au sevrage tabagique
- Instaurer une activité physique régulière

Dans le but d’offrir une solution complète de prise en charge à nos patients, nous avons pensé à mettre en place une plateforme digitale qui vise à surveiller le patient à distance et renforcer ses capacités du malade de gérer sa pathologie au quotidien.

Il s’agit d’un projet innovant dans notre contexte tunisien qui propose de nouveaux modes de surveillance et d’accompagnement thérapeutique aux patients souffrant d’une pathologie cardiovasculaire. En effet, il s’agit de la 1ère plateforme digitale de télé-suivi et télé-accompagnement thérapeutique en Tunisie en pathologie cardio-vasculaire.

Cet outil digital constitue une innovation à la fois pour les patients qui se voient proposer un programme de surveillance et d’apprentissage personnalisé et adapté à leur profil et leurs souhaits ; mais aussi pour les professionnels de santé qui ont désormais à leur disposition un outil de suivi des objectifs du patient et des modules ludiques d’apprentissage de connaissance.

Cette expérience pilote pourrait être généralisée à plusieurs services et à plusieurs disciplines. Elle pourrait être utilisée dans plusieurs travaux de recherche.

Méthodologie

# I. Type et lieu d’étude :

Cette étude est un essai clinique contrôlé. Elle sera menée entre mai 2022 et décembre 2023. Au total, 100 patients atteints de maladie coronarienne seront recrutés parmi les patients hospitalisés dans le service de cardiologie du CHU Sahloul et seront répartis de manière égale en deux groupes : le groupe d'intervention et un groupe témoin.

# II. population d’étude

## Au total, 100 patients atteints de maladie coronarienne seront recrutés parmi les patients hospitalisés dans le service de cardiologie du CHU Sahloul et seront répartis de manière égale en deux groupes : le groupe d'intervention et un groupe témoin.

## Critères d’inclusion

Ce programme est destiné aux patients coronariens et leurs entourages :

-	Patient venant de présenter un syndrome coronarien aigu

-	Patient revascularisé (angioplastie ou pontage coronarien)

-	Patient angineux stable ou asymptomatique (ischémie silencieuse)

# III. déroulement de l’etude

## 1. Déroulement de l’intervention

### Au total, 100 patients atteints de maladie coronarienne seront recrutés sur un site clinique et répartis de manière égale en deux groupes : le groupe d'intervention et un groupe témoin. Les patients affectés au groupe d'intervention recevront d'abord une éducation thérapeutique. La plateforme numérique permettra ensuite aux prestataires de soins de les accompagner à distance.

Le suivi des patients seront faits pour les deux groupes à 3, et 12 mois.

Tout d’abord, les patients des deux groupes vont répondre à un questionnaire pour évaluer leurs connaissances et pour mener le diagnostic éducatif du groupe intervention.

A 3 mois après la réalisation du programme d’ETP, le même questionnaire sera distribué aux deux groupes, un bilan biologique (Hémoglobine glyquée, TG, Cholestérol total, LDL, HDL) sera demandé et une évaluation de la qualité de vie sera faite.

A 12 mois, une évaluation du maintien des connaissances sera réalisée, ainsi que la recherche de l’incidence des événements cardiovasculaires majeurs.

Dans un deuxième temps, le groupe intervention va bénéficier d’un suivi à distance à travers une plateforme digitale.

Le groupe témoin sera suivi seulement dans la consultation externe de cardiologie.

## 2. Critère de jugement

- **Critère de jugement principal** : l'incidence des événements cardiovasculaires majeurs dans l'année qui suit la sortie de l'hôpital pour le groupe contrôle et dans l’année qui suit la réalisation du programme d’ETP avec suivi à distance pour le groupe intervention.
- **Critères secondaires** sont les changements dans les comportements de santé (arrêt de tabac, adoption d’un régime alimentaire sain, pratique de l’activité physique) et l’évaluation de la qualité de vie.

## 3. Programme d’éducation thérapeutique :

Un questionnaire sera distribué aux patients avant de commencer le programme d’ETP évaluant :

- Leurs connaissances sur la maladie,

- Leurs habitudes alimentaires,

- Leurs activités physiques,

Le même questionnaire sera distribué une autre fois en fin de l’intervention afin d’étudier l’impact du programme d’ETP en évaluant les modifications des comportements.

Les séances d’éducation seront réalisées à raison d’une séance chaque deux semaines. Chaque séance sera répétée quatre fois tout au long de la période d’étude.

## 4. La constitution de l’équipe pluridisciplinaire :

L’ETP doit faire appel à des professionnels formés à la fois à l’éducation et à la pathologie coronarienne. L’implication des cardiologues est essentielle pour l’animation et la coordination des programmes.

Les différents intervenants devront apprendre à utiliser un langage commun et à proposer des réponses homogènes aux patients au cours des différentes séances éducatives.

Un informaticien va assister aux différentes séances afin de pouvoir traduire les différentes tâches menées en programme informatisé.

Ainsi, l’équipe responsable de l’ETP de la maladie coronarienne est composée de :

- **Médecin cardiologue** : Responsable de faire le diagnostic de la maladie et l’annoncer aussi au patient que son entourage proche. Il va aussi expliquer les principes anatomiques et physiopathologiques de la maladie.
- **Diététicienne** : Son rôle est d’accompagner les malades dans leur régime alimentaire tout en promouvant l’alimentation cardio-protectrice et promouvoir l’activité physique.
- **Tabacologue** : Responsable de l’aide au sevrage tabagique pour tous ceux qui fument et de la sensibilisation aux méfaits du tabac sur la santé en général et sur la maladie coronarienne en particulier.
- **Pour la solution digitale** :
- Club de télémédecine de la faculté de médecine de Sousse
- DACIMA consulting

Chaque membre de l’équipe doit garder son identité professionnelle tout en travaillant en interdépendance, et avoir la compétence en communication dans le domaine de la santé.

En effet, la communication professionnelle est une forme particulière de communication interpersonnelle. Dans le domaine de la santé, elle se définit comme un entretien, verbal ou autre, entre un professionnel de la santé, le patient, les proches de celui-ci, un collègue ou un public (11). Dans une relation professionnelle, une communication interpersonnelle réussie suppose une coopération entre les participants.

## 5. Dossier Education Thérepeutique de Patient :

Chaque patient aura un dossier ETP personnalisé.

Ce dossier sera informatisé permettant à chaque membre de l’équipe responsable d’y accéder pour actualiser les données sur chaque patient et son progrès dans le parcours d’ETP.

Le dossier comportera :

- le contrat de consentement de participation au programme ETP
- les données sociodémographiques du patient
- les caractéristiques cliniques et les antécédentes médicaux
- le diagnostic éducatif
- le parcours réalisé par le patient
- l’évaluation individuelle du patient.

## 6. Les outils éducatifs :

Pour notre projet d’éducation thérapeutique des patients coronariens, on a choisi les outils suivants :

- **Une séquence vidéo** : Il s’agit d’un enregistrement d’un jeu de rôle entre un médecin et un patient simulé présentant un entretien motivationnel ayant pour but de déplacer le patient du stade de «contemplation » vers le stade de « préparation ». Cet enregistrement a été fait en deux langues : arabe tunisien (le Darija) et français.

Lien : Version Arabe tunisien : <https://youtu.be/RD4GqO90j1w>

Version Française : <https://youtu.be/X8w7AXpHBY0>

- **Une brochure** : Une brochure en arabe sera fournie aux patients lors des séances éducatives expliquant les méfaits du taux élevé de cholestérol ainsi que les aliments susceptibles de diminuer les taux de cholestérol dans le sang.
- **Planches anatomiques** : Qui illustrent la physiopathologie de la lésion thrombo-athéromateuse et les principes de l’angioplastie coronaire.
- **Photos** : Des photos seront utilisés pour les différents ateliers sur le régime alimentaire, qui fournissent les détails nécessaires sur l’alimentation cardio-protectrice, sur les aliments pauvres en sel ainsi que des conseils diététiques.

L’OMS a défini l’ETP comme étant un processus continu, intégré dans les soins et centré sur le patient. Il comprend des activités organisées de sensibilisation, d’information, d’apprentissage et d’accompagnement psychosocial concernant la maladie, le traitement prescrit, les soins, l’hospitalisation et les autres institutions de soins concernées, et les comportements de santé et de maladie du patient (12).

Il s’agit d’une démarche éducative qui repose sur un programme structuré, organisé et centré sur le patient (12 – 14).

De ce fait, l’ETP part d’une logique de compétences pour aboutir à des objectifs terminaux d’intégration. Ce mixage de la pédagogie par objectifs et de la logique de compétences est au cœur du programme éducatif personnalisé (15).

Le référentiel de compétences des patients intervenants est fondé sur l’approche par compétence, l’apprentissage et la construction d’un savoir agir professionnel permettant au patient de développer/d’acquérir une identité professionnelle à part entière dans le champ de l’ETP.

L’Approche par compétence est une formation qui part d’une situation authentique où l’apprenant est au centre, accompagné par son formateur dans le but d’évoluer vers les attendus de cette formation. A partir de cette définition, on peut dégager les repères d’une compétence qui sont :

- Centrage sur l’apprenant
- Accompagnement
- Situation authentique contextualisée
- Progression de la formation dans le temps

Et ils s’ajoutent :

- Evaluation des formateurs par les patients et une auto-évaluation
- Autonomie

Dans cette approche, l’ETP, de par sa nature et ses caractéristiques, n’est pas directement assimilable à un acte de soins, ce qui signiﬁe que l’expérience et les méthodes acquises en matière de caractérisation des actes et techniques de soins ne sont pas transposables à cette pratique.

Afin de mener notre programme d’éducation thérapeutique, nous allons suivre les étapes comme élaborées par la Haute autorité de santé (HAS) (13).

## 7. Etape du programme de l’Education Thérapeutique du Patient :

### a. Diagnostic éducatif :

Le programme débute par un « Diagnostic Educatif », appelé également « bilan éducatif partagé » qui est réalisé par des entretiens individuels semi-directifs. Cette étape est indispensable à l’identification des besoins et des attentes du patient, et sert aussi à identifier les freins et les leviers permettant d’assurer le succès de l’ETP.

Elle permet de formuler avec lui les compétences à acquérir ou à mobiliser.

Le diagnostic éducatif cherche à mettre en adéquation les priorités du patient et celles des soignants en élaborant des objectifs éducatifs négociés.

Le guide d’entretien est élaboré à partir des données de la littérature (13, 16 – 19).

Cet entretien sera mené dans une ambiance conviviale, tout en évaluant le degré de motivation du patient.

### b. Contrat éducatif et Définition du programme personnalisé d’Education Thérapeutique du Patient :

Le diagnostic aboutit à la définition des compétences que le patient est amené à acquérir grâce aux rencontres avec les professionnels de santé (20).

Les compétences à acquérir sont négociées avec le patient au sein « d’un contrat d’éducation». Elles correspondent à un ensemble de connaissances, d’actions ou de comportements que le patient devrait maîtriser pour gérer son traitement et prévenir la survenue des complications tout en maintenant ou en améliorant sa qualité de vie (21).

Elles doivent être réalistes et accessibles.

### c. L’évaluation des compétences acquises et du déroulement du programme :

L’évaluation de programme vise à améliorer le processus d’ETP : sa conception, son organisation, sa réalisation, son évaluation.

Elle sert à pratiquer des réajustements ou des réorientations des pratiques professionnelles. En éducation thérapeutique, évaluer c’est collecter des informations pertinentes, fiables, valides, les analyser et les comparer à des éléments de référence ou normes.

Nous proposons 3 grilles d’évaluation :

- Grille d’évaluation d’une séance éducative individuelle
- Grille d’évaluation d’une séance éducative collective
- Grille d’auto-évaluation annuelle
- Evaluation de l’impact des séances d’ETP.

L’auto-évaluation annuelle est élaborée en se référant au guide de la HAS pour l’élaboration de la grille d’auto-évaluation annuelle d’un programme d’éducation thérapeutique (24).

Elle porte sur les éléments suivants :

- Une auto-évaluation de l’activité globale du programme à partir d’un questionnement et d’une analyse de données qualitatives et quantitatives au regard des objectifs du programme d’ETP. Ces données, concernant notamment les patients engagés dans le programme, la contribution des intervenants, les activités éducatives, le partage des informations, etc.

- Une auto-évaluation du processus du programme à partir d’un questionnement sur les façons de procéder et l’enchaînement des étapes de l’ETP. Leur analyse permet d’identifier les différences éventuelles avec ce que l’équipe a prévu dans le programme d’ETP et de les expliquer.

- Une auto-évaluation de l’atteinte des objectifs du programme à partir d’un questionnement et d’une analyse de l’écart entre ce que le programme a prévu d’atteindre et les changements observés chez les bénéficiaires et les intervenants dans le programme.

Cette évaluation est basée sur les quatre étapes de la HAS

- Choix des objets d’évaluation
- Recueil des données
- Identification des forces, des faiblesses, des difficultés de mise en œuvre du programme et hiérarchisation des propositions d’améliorations
- Mise en œuvre des actions d’amélioration

## 8. Description de la solution digitalique

1- **La télé-éducation thérapeutique**

L’accès aux outils éducatifs (supports numériques) est permis à tous les adhérents initiaux au programme. Une mise à jour de ces outils sera effectuée par l’équipe de réadaptation de façon annuelle selon les recommandations des sociétés savantes.

La plateforme offre aussi la possibilité d’organisation d’une séance éducative à distance individuelle ou collective (modules de visioconférence permettant d'effectuer des séances collectives et individuelles), une messagerie sécurisée intégrée soignants-patients

2- **Modalités d'interventions et d'organisation proposées**

Le parcours patient en télé-surveillance et télé-accompagnement thérapeutique est ainsi détaillé :

- **Etape 1 : Inclusion à la télé-surveillance et télé-éducation.**

A la fin du programme de réadaptation cardiaque ambulatoire (phase II), l'équipe de réadaptation vérifie les conditions d'éligibilité au programme de télé-surveillance et télé-éducation. Un contrat « d’accompagnement thérapeutique » est signé par l’adhérant au programme et l’équipe de réadaptation dans lequel sont fixés les objets thérapeutiques pendant le suivi ainsi que les paramètres à recueillir et à transmettre à la plateforme.

L'équipe de réadaptation l'éduque à l'utilisation de l'application mobile qui reprend les codes et usages des applications grand public et l'éduque à l'utilisation des objets connectés.

Durant le séjour de réadaptation à domicile, le patient peut accéder à un FAQ ou à une assistance téléphonique en cas de besoin.

- **Etape 2 : Phase de télé-surveillance.**

Chaque jour, le patient est invité à transmettre les paramètres de suivi fixés par le contrat « d’accompagnement thérapeutique »

La transmission des données sera faite de manière asynchrone et sécurisée sur la plateforme de suivi.

- **Etape 3 : Phase d’analyse des données**

L'équipe médicale peut interagir avec le patient via la plateforme. Si besoin, le patient peut être convoqué en centre par l'équipe médicale pour réévaluation et consultation anticipée afin d'optimiser son parcours de soins. (en cas d’alerte rouge par exemple)

- **Etape 4 : Phase d’évaluation**

L'équipe réalise l'évaluation clinique et para clinique systématique à 6 mois, à 12 mois et 18 mois. Les résultats intermédiaires et finaux seront publiés au fur et à mesure.

# IV. Analyse statistique :

Les données seront analysées à l'aide du logiciel statistique IBM SPSS version 22.0 (SPSS Inc., Chicago, IL, USA).

- La distribution normale et l'égalité de variance seront évaluées avant toute analyse statistique à l'aide du test de Kolmogorov-Smirnov.

- Une analyse descriptive sera effectuée avec la moyenne, l'écart-type et les valeurs minimales et maximales. Pour les variables à distribution non-normale, la médiane sera utilisée comme mesure de centralisation et les percentiles 25 et 75 comme mesures de dispersion.

Les fréquences absolues et relatives seront données pour les variables qualitatives.

Pour les données quantitatives, la comparaison entre deux moyennes sera effectuée à l'aide du test t de Student ou test U de Mann-Whitney.

Le test Chi2 de Pearson et le test exact de Fisher seront utilisés pour la comparaison des porcentages.

Le seuil de significativité sera fixé à 0,05.

# V. Considérations éthiques :

Le protocole de l'étude sera soumis à l'approbation du comité d'éthique de l'hôpital universitaire Sahloul, Sousse, Tunisie.

Tous les participants vont signer un consentement écrit.

L'investigateur présentera les objectifs de l'étude et assurera également la confidentialité et l'anonymat des données lors des analyse.

- Les patients seront libres de participer à l'étude.

- Confidentialité des données :

Les données seront collectées par les médecins du projet et traitées dans le département de prévention et de sécurité des soins de manière anonyme.

Références

1- Rouseff M, Aneni EC, Guzman H, Das S, Brown D, et al. One-year outcomes of an intense workplace cardio-metabolic risk reduction program among high-risk employees: The My Unlimited Potential. Obes Silver Spring Md. 2016 Jan;24(1):71–8.
2- Chen J, Wu X, Gu D. Hypertension and cardiovascular diseases intervention in the capital steel and iron company and Beijing Fangshan community. Obes Rev. 2008 Mar 1;9:142–5.
3- World Health Organization. Cardiovascular diseases (CVDs) [Internet]. Geneva 2017 Accessible sur: <http://www.who.int/mediacentre/factsheets/fs317/en/> (Consulté le 10/05/2019)
4- OMS : Les maladies cardiaques, première cause de mortalité en Tunisie. 2014. Accessible sur : <https://www.realites.com.tn/2014/09/oms-les-maladies-cardiaques-premiere-cause-de-mortalite-en-tunisie/> (Consulté le 03/12/2018)
5- Pavy B, Barbet R, Carré F, Champion C, Iliou MC, et al. Education thérapeutique dans la maladie coronarienne : position du GERS (groupe exercice, réadaptation et sport) et de la commission d’éducation thérapeutique de la société française de cardiologie. 2013. Accessible sur : <http://www.sfcardio.fr/sites/default/files/pdf/ETP_maladiecoronaire-222.pdf> (Consulté le 29/10/2028).
6- Wood DA, Kotseva K, Connolly S, Jennings C, Mead A, et al. Nurse-coordinated multidisciplinary, family-based cardiovascular disease prevention programme (EUROACTION) for patients with coronary heart disease and asymptomatic individuals at high risk of cardiovascular disease: a paired, cluster-randomized controlled trial. Lancet. 2008;371:1999-2012.
7- Giannuzzi P, Temporelli PL, Marchioli R, Maggioni AP, Balestroni G, et al. Global secondary prevention strategies to limit event recurrence after myocardial infarction: results of the GOSPEL study, a multicenter, randomized controlled trial from the Italian Cardiac Rehabilitation network. Arch Int Med. 2008;168(20):2194-204.
8- Therapeutic patient education. Continuing education programmes for health care providers in the field of prevention of chronic diseases. Report of a WHO working group. Copenhagen, WHO Regional Office for Europe, 1998. Accessible sur : <http://www.euro.who.int/__data/assets/pdf_file/0007/145294/E63674.pdf> (Consulté le 03/12/2018).
9- Lasserre Moutet A. Educateur ou psychothérapeute : où se situe la frontière ? DELF 2008.
10- Annamaud Roussel. Évaluation de l’efficacité d’un programme d’éducation thérapeutique chez les insuffisants cardiaques : le programme I-Care à l’hôpital Saint-André de Bordeaux. Médecine humaine et pathologie. 2015. ffdumas-01117411f
11- Richard C, Lussier MT. La communication professionnelle en santé. ERPI Médecine (2^ème^ édition).
12- Programmes de formation continue pour professionnels de soins dans le domaine de la prévention des maladies chroniques. Recommandations d'un groupe de travail de l'O.M.S. 1998. Accessible sur : <http://www.euro.who.int/__data/assets/pdf_file/0009/145296/E93849.pdf>
13- HAS. Éducation thérapeutique du patient Comment la proposer et la réaliser ? Juin 2007. Accessible sur : [www.has-sante.fr](http://www.has-sante.fr)
14- HAS. Guide méthodologique: Structuration d’un programme d’éducation thérapeutique du patient dans le champ des maladies chroniques. HAS; 2007. Accessible sur : [www.has-sante.fr](http://www.has-sante.fr)
15- Chauvigné C, Coulet JC. L’approche par compétences : un nouveau paradigme pour la pédagogie universitaire ? Revue française en Pédagogie. 2010 :15-28.
16- HAS : Education thérapeutique du patient. Définition, finalités et organisations. Juin 2007.
17- Sandrin-Berthon B. Diagnostique éducatif ou bilan éducatif partagé ? Médecine des maladies métaboliques, Edition Masson.2010;4(1):38-43.
18- Simon D, Traynard PY, Bourdillon, Grimaldi A. Education thérapeutique : prévention et maladies chroniques. Elsevier Masson éd, Issy-les Moulineaux 2007, 269 pages.
19- D’Ivernois JF, Gagnayre R. Apprendre à éduquer le patient. Maloine éd, Paris 2004, 155 pages.
20- Les étapes de la démarche éducative. Argumentaire. Rev Mal Respir.2002; 19:2S59-2S75. SPLF, Paris 2002.
21- World Health Organization. Therapeutic patient education: continuing education programmes for healthcare providers in the field of prevention of chronic diseases. Copenhaguen: WHO; 1998.
22- National Institutes of health, National Heart Lung and Blood Institute. Guidelines for the diagnosis and management of asthma. Bethesda (MD): *NIH*; 1997.
23- National Heart Lung and Blood Institute. Organisation mondiale de la santé. Vaincre l’asthme : stratégie globale pour la guidance de l’asthme et sa prévention. *Rev FR Allergo Imminol Clin.* 1996 ;(36)6.
24- Évaluation annuelle d’un programme d’éducation thérapeutique du patient (ETP) : une démarche d'auto-évaluation. Guide méthodologique pour les coordonnateurs. HAS. Mai 2014. Accessible sur : <https://www.has-sante.fr/portail/jcms/c_1234324/fr/evaluation-annuelle-d-un-programme-d-education-therapeutique-du-patient-etp-une-demarche-d-auto-evaluation>
