# Supplementary material for: Evaluation of a therapeutic education program for patients with coronary heart disease in a Tunisian university hospital: A quasi-experimental study
Source: PLoS One. 2026 Nov 9;21(11):e0361814. doi: 10.1371/journal.pone.0361814 (PMC13747245; doi:10.1371/journal.pone.0361814)
Supplement: Supplementary file 4 [file pone.0361814.s005.docx]

**Therapeutic education of coronary patients: creation of a digital monitoring platform**

**Dr. Hela Ghali**

Introduction

Cardiovascular diseases (CVD) are a major global health problem (1,2). According to the World Health Organization (WHO), CVD was responsible for 17.5 million deaths in 2012 and more than three-quarters of CVD deaths occurred in low- and middle-income countries (3).

According to a recent report published by the WHO, cardiovascular diseases are the main cause of death in Tunisia, far ahead of infectious diseases (4).

Stronger than HIV, cancers or even diabetes, cardiovascular diseases constitute the leading cause of mortality in Tunisia with 49% of all deaths.

Faced with the increase in the number of chronic diseases with the aging of the population, and with the observation of the inadequacy of therapeutic control, a new need has emerged, that of having a patient as a care partner.

Therapeutic education is a humanistic approach centered on the patient, his needs and his resources. It is proposed not only to help the patient understand his illness and his treatment but also to help him become autonomous.

The management of modifiable risk factors remains a priority, both in primary and secondary prevention, to improve the prognosis of this formidable condition whose asymptomatic nature is not synonymous with benignity. Assistance with smoking cessation, regular physical activity, a cardio-protective diet (the "Mediterranean" diet), stress management, good adherence to drug treatment which improves its observance, judicious use of the health system, assistance with professional reintegration are the current challenges of therapeutic patient education (TPE) for coronary patients (5).

The interest of TPE in coronary patients has been highlighted by several types of approaches.

However, in most therapeutic education programs, patient monitoring stops at the end of the education program. However, it is essential to support the coronary patient in the third phase of their care. Thus, our study aims to create a digital platform allowing patient monitoring after having benefited from a therapeutic education program.

In this context, we have chosen to plan a therapeutic education program for coronary patients consulting the cardiology department of Sahloul University Hospital, in collaboration with the Prevention and Safety of Care department.

The TP sessions aim to provide coronary patients with the means to maintain skills to improve their health and quality of life and to make them responsible for managing their disease, in order to live better with their disease. Thus, the objectives will be:

- Improving patient compliance
- Adopt healthy eating behavior
- Help with smoking cessation
- Establish regular physical activity

In order to offer a complete care solution to our patients, we thought about setting up a digital platform which aims to monitor the patient remotely and strengthen the patient's ability to manage their pathology on a daily basis.

This is an innovative project in our Tunisian context that offers new methods of monitoring and therapeutic support to patients suffering from cardiovascular disease. Indeed, it is the first digital platform for remote monitoring and therapeutic remote support in Tunisia in cardiovascular disease.

This digital tool is an innovation both for patients who are offered a personalized monitoring and learning program adapted to their profile and wishes; but also for health professionals who now have at their disposal a tool for monitoring the patient's objectives and fun knowledge learning modules.

This pilot experience could be generalized to several services and several disciplines. It could be used in several research works.

Methodology

# I. Type and location of study:

This study is a controlled clinical trial. It will be conducted between May 2022 and December 2023. A total of 100 patients with coronary heart disease will be recruited from among the patients hospitalized in the cardiology department of Sahloul University Hospital and will be divided equally into two groups: the intervention group and a control group.

# II. study population

## A total of 100 patients with coronary heart disease will be recruited from among patients hospitalized in the cardiology department of Sahloul University Hospital and will be divided equally into two groups: the intervention group and a control group.

## Inclusion criteria

This program is intended for coronary patients and their families:

-	Patient who has just presented with acute coronary syndrome

-	Revascularized patient (angioplasty or coronary bypass)

-	Stable or asymptomatic angina patient (silent ischemia)

# III. progress of the study

## 1. Progress of the intervention

### A total of 100 patients with coronary artery disease will be recruited at a clinical site and divided equally into two groups: the intervention group and a control group. Patients assigned to the intervention group will first receive therapeutic education. The digital platform will then allow healthcare providers to support them remotely.

Patient follow-up will be done for both groups at 3 and 12 months.

First, patients in both groups will answer a questionnaire to assess their knowledge and to conduct the educational diagnosis of the intervention group.

Three months after the ETP program has been completed, the same questionnaire will be distributed to both groups, a biological assessment (glycated hemoglobin, TG, total cholesterol, LDL, HDL) will be requested and an assessment of the quality of life will be made.

At 12 months, an assessment of knowledge maintenance will be carried out, as well as research into the incidence of major cardiovascular events.

In a second phase, the intervention group will benefit from remote monitoring through a digital platform.

The control group will be followed only in the cardiology outpatient clinic.

## 2. Judgment criterion

- **Primary judgment criterion**: the incidence of major cardiovascular events in the year following hospital discharge for the control group and in the year following completion of the ETP program with remote monitoring for the intervention group.
- Secondary endpoints are changes in health behaviors (smoking cessation, adoption of a healthy diet, physical activity) and assessment of quality of life.

## 3. Therapeutic education program:

A questionnaire will be distributed to patients before starting the TPE program assessing:

- Their knowledge about the disease,

- Their eating habits,

- Their physical activities,

The same questionnaire will be distributed again at the end of the intervention to study the TPE program's impact by evaluating behavior changes.

The education sessions will be conducted at a rate of one session every two weeks. Each session will be repeated four times throughout the study period.

## 4. The constitution of the multidisciplinary team:

The ETP must involve professionals trained in both education and coronary pathology. The involvement of cardiologists is essential for the animation and coordination of programs.

The various stakeholders will have to learn to use a common language and to offer consistent responses to patients during the various educational sessions.

A computer scientist will attend the various sessions in order to be able to translate the different tasks carried out into a computer program.

Thus, the team responsible for the ETP of coronary disease is composed of:

- **Cardiologist**: Responsible for diagnosing the disease and announcing it to the patient and those close to him. He will also explain the anatomical and pathophysiological principles of the disease.
- **Dietician**: Its role is to support patients in their diet while promoting cardio-protective nutrition and promoting physical activity.
- **Tabacologist**: Responsible for helping all smokers quit smoking and raising awareness of the harmful effects of tobacco on health in general and coronary heart disease in particular.
- **For the digital solution**:
- Telemedicine Club of the Faculty of Medicine of Sousse
- DACIMA consulting

Each team member must maintain their professional identity while working interdependently and have communication skills in the health field.

Indeed, professional communication is a form of interpersonal communication. In the health field, it is defined as a conversation, verbal or otherwise, between a health professional, the patient, the patient's relatives, a colleague or an audience (11). In a professional relationship, successful interpersonal communication requires cooperation between the participants.

## 5. Patient Therapeutic Education File:

Each patient will have a personalized TPE file.

This file will be computerized, allowing each member of the responsible team to access it to update the data on each patient and their progress in the TPE pathway.

The file will include:

- the TPE program participation consent contract
- patient's sociodemographic data
- clinical features and medical history
- educational diagnosis
- the journey taken by the patient
- individual patient assessment.

## 6. Educational tools:

For our therapeutic education project for coronary patients, we have chosen the following tools:

- **A video sequence**: This is a recording of a role play between a doctor and a simulated patient presenting a motivational interview with the aim of moving the patient from the stage of "contemplation" to the stage of "preparation". This recording was made in two languages: Tunisian Arabic (Darija) and French.

Link: Tunisian Arabic version:<https://youtu.be/RD4GqO90j1w>

French version:<https://youtu.be/X8w7AXpHBY0>

- **A brochure**: A brochure in Arabic will be provided to patients during the educational sessions explaining the harmful effects of high cholesterol as well as foods that can lower blood cholesterol levels.
- **Anatomical charts**: Which illustrate the pathophysiology of thrombo-atheromatous lesion and the principles of coronary angioplasty.
- **Photos**: Photos will be used for the various diet workshops, which provide necessary details on cardio-protective diet, low-salt foods as well as dietary advice.

WHO has defined TPE as a continuous, integrated, patient-centred process that includes organized awareness-raising, information, learning and psychosocial support activities regarding the disease, the prescribed treatment, care, hospitalization and other relevant care institutions, and the patient's health and illness behaviours (12).

It is an educational approach based on a structured, organized and patient-centered program (12 – 14).

As a result, TPE starts from a skills-based logic to achieve terminal integration objectives. This mix of pedagogy by objectives and skills-based logic is at the heart of the personalized educational program (15).

The skills framework for patient interveners is based on the skills-based approach, learning and the construction of professional knowledge allowing the patient to develop/acquire a full-fledged professional identity in the field of TPE.

The Competency-Based Approach is a training that starts from an authentic situation where the learner is at the center, accompanied by his trainer to evolve towards the expectations of this training. From this definition, we can identify the benchmarks of a skill which are:

- Learner-centeredness
- Accompaniement
- Authentic situation contextualized
- Progression of training over time

And they add:

- Patient evaluation of trainers and self-assessment
- Autonomy

In this approach, TPE, by its nature and characteristics, is not directly comparable to an act of care, which means that the experience and methods acquired in terms of characterization of acts and techniques of care are not transposable to this practice.

In order to carry out our therapeutic education program, we will follow the steps as developed by the High Authority for Health (HAS) (13).

## 7. Step of the Patient Therapeutic Education program:

### a. Educational diagnosis:

The program begins with an "Educational Diagnosis", also called a "shared educational assessment" which is carried out through semi-directed individual interviews. This step is essential to identify the patient's needs and expectations and also serves to identify the obstacles and levers to ensure the success of the TPE.

It allows you to formulate with him the skills to be acquired or mobilized.

Educational diagnosis seeks to align the priorities of the patient and those of the caregivers by developing negotiated educational objectives.

The interview guide is developed based on data from the literature (13, 16 – 19).

This interview will be conducted in a friendly atmosphere, while assessing the patient's level of motivation.

### b. Educational contract and Definition of the personalized Patient Therapeutic Education program:

The diagnosis results in the definition of the skills that the patient is expected to acquire through meetings with health professionals (20).

The skills to be acquired are negotiated with the patient within an "education contract". They correspond to a set of knowledge, actions or behaviors that the patient should master to manage his treatment and prevent the occurrence of complications while maintaining or improving his quality of life (21).

They must be realistic and accessible.

### c. Assessment of acquired skills and program progress:

Program evaluation aims to improve the TPE process: its design, organization, implementation, evaluation.

It is used to carry out readjustments or reorientations of professional practices. In therapeutic education, evaluation means collecting relevant, reliable, valid information, analyzing it and comparing it to reference elements or standards.

We offer 3 evaluation grids:

- Evaluation grid for an individual educational session
- Evaluation grid for a group educational session
- Annual self-assessment grid
- Evaluation of the impact of TPE sessions.

The annual self-assessment is developed with reference to the HAS guide for developing the annual self-assessment grid for a therapeutic education program (24).

It covers the following elements:

- A self-assessment of the overall program activity based on questioning and analyzing qualitative and quantitative data regarding the objectives of the TPE program. This data, in particular, concerns patients involved in the program, the contribution of stakeholders, educational activities, information sharing, etc.

- A self-assessment of the program process based on a questioning of the ways of proceeding and the sequence of the TPE steps. Their analysis makes it possible to identify any differences with what the team has planned in the TPE program and to explain them.

- A self-assessment of the achievement of the program objectives based on questioning and an analysis of the gap between what the program planned to achieve, and the changes observed among beneficiaries and stakeholders in the program.

This assessment is based on the four stages of the HAS

- Selection of evaluation objects
- Data collection
- Identification of strengths, weaknesses, difficulties in implementing the program and prioritization of improvement proposals
- Implementation of improvement actions

## 8. Description of the digital solution

1- **Therapeutic tele-education**

Access to educational tools (digital media) is allowed to all initial members of the program. An update of these tools will be carried out by the rehabilitation team on an annual basis according to the recommendations of learned societies.

The platform also offers the possibility of organizing an individual or collective remote educational session (videoconferencing modules allowing collective and individual sessions), an integrated secure caregiver-patient messaging service

2- **Proposed intervention and organizational modalities**

The patient pathway in remote monitoring and therapeutic remote support is detailed as follows:

- **Step 1: Inclusion in remote monitoring and remote education.**

At the end of the outpatient cardiac rehabilitation program (phase II), the rehabilitation team checks the eligibility conditions for the telemonitoring and teleeducation program. A "therapeutic support" contract is signed by the program member and the rehabilitation team in which the therapeutic objects during monitoring are set out as well as the parameters to be collected and transmitted to the platform.

The rehabilitation team teaches him how to use the mobile application, which uses the codes and practices of consumer applications, and teaches him how to use connected objects.

During the home rehabilitation stay, the patient can access a FAQ or telephone assistance if needed.

- **Step 2: Remote monitoring phase.**

Every day, the patient is asked to transmit the monitoring parameters set by the “therapeutic support” contract.

Data transmission will be done asynchronously and securely on the tracking platform.

- **Step 3: Data analysis phase**

The medical team can interact with the patient via the platform. If necessary, the patient can be summoned to the center by the medical team for reassessment and early consultation in order to optimize their care pathway. (in the event of a red alert for example)

- **Step 4: Evaluation phase**

The team performs systematic clinical and paraclinical assessment at 6 months, 12 months and 18 months. Intermediate and final results will be published as they occur.

# IV. Statistical analysis:

Data will be analyzed using IBM SPSS statistical software version 22.0 (SPSS Inc., Chicago, IL, USA).

- Normal distribution and equality of variance will be assessed before any statistical analysis using the Kolmogorov-Smirnov test.

- A descriptive analysis will be performed with the mean, standard deviation and minimum and maximum values. For variables with non-normal distribution, the median will be used as a measure of centralization and the 25th and 75th percentiles as measures of dispersion.

Absolute and relative frequencies will be given for qualitative variables.

For quantitative data, the comparison between two means will be made using the Student t-test or Mann-Whitney U test.

Pearson Chi2 test and Fisher exact test will be used for comparison of percentages.

The significance threshold will be set at 0.05.

# V. Ethical considerations:

The study protocol will be submitted for approval to the ethics committee of Sahloul University Hospital, Sousse, Tunisia.

All participants will sign a written consent.

The investigator will present the objectives of the study and will also ensure the confidentiality and anonymity of the data during analysis.

- Patients will be free to participate in the study.

- Data confidentiality:

The data will be collected by the project doctors and processed in the prevention and safety of care department anonymously.

References

1- Rouseff M, Aneni EC, Guzman H, Das S, Brown D, et al. One-year outcomes of an intense workplace cardio-metabolic risk reduction program among high-risk employees: The My Unlimited Potential. Obes Silver Spring Md. 2016 Jan;24(1):71–8.
2- Chen J, Wu X, Gu D. Hypertension and cardiovascular diseases intervention in the capital steel and iron company and Beijing Fangshan community. Obes Rev. 2008 Mar 1;9:142–5.
3- World Health Organization. Cardiovascular diseases (CVDs) [Internet]. Geneva 2017 Accessible on:<http://www.who.int/mediacentre/factsheets/fs317/en/>(Accessed 05/10/2019)
4- WHO: Heart disease, leading cause of death in Tunisia. 2014. Available at:[https://www.realites.com.tn/2014/09/oms-les-entreprises-cardiaques-premiere-cause-de-mortalite-en-tunisie/](https://www.realites.com.tn/2014/09/oms-les-maladies-cardiaques-premiere-cause-de-mortalite-en-tunisie/)(Accessed 03/12/2018)
5- Pavy B, Barbet R, Carré F, Champion C, Iliou MC, et al. Therapeutic education in coronary artery disease: position of the GERS (exercise, rehabilitation and sport group) and the therapeutic education commission of the French society of cardiology. 2013. Available on:[http://www.sfcardio.fr/sites/default/files/pdf/ETP_morbidecoronaire-222.pdf](http://www.sfcardio.fr/sites/default/files/pdf/ETP_maladiecoronaire-222.pdf)(Accessed 29/10/2028).
6- Wood DA, Kotseva K, Connolly S, Jennings C, Mead A, et al. Nurse-coordinated multidisciplinary, family-based cardiovascular disease prevention program (EUROACTION) for patients with coronary heart disease and asymptomatic individuals at high risk of cardiovascular disease: a paired, cluster-randomized controlled trial. Lancet. 2008;371:1999-2012.
7- Giannuzzi P, Temporelli PL, Marchioli R, Maggioni AP, Balestroni G, et al. Global secondary prevention strategies to limit event recurrence after myocardial infarction: results of the GOSPEL study, a multicenter, randomized controlled trial from the Italian Cardiac Rehabilitation network. Arch Int Med. 2008;168(20):2194-204.
8- Therapeutic patient education. Continuing education programs for health care providers in the field of prevention of chronic diseases. Report of a WHO working group. Copenhagen, WHO Regional Office for Europe, 1998. Accessible on:<http://www.euro.who.int/__data/assets/pdf_file/0007/145294/E63674.pdf>(Accessed 03/12/2018).
9- Lasserre Moutet A. Educator or psychotherapist: where is the boundary? DELF 2008.
10- Annamaud Roussel. Evaluation of the effectiveness of a therapeutic education program for heart failure patients: the I-Care program at Saint-André hospital in Bordeaux. Human medicine and pathology. 2015. ffdumas-01117411f
11- Richard C, Lussier MT. Professional communication in health. ERPI Medicine (2nd edition).
12- Continuing education programmes for health care professionals in the field of chronic disease prevention. Recommendations of a WHO working group 1998. Available at:<http://www.euro.who.int/__data/assets/pdf_file/0009/145296/E93849.pdf>
13- HAS. Therapeutic education of the patient How to propose and carry it out? June 2007. Accessible on:[www.has-sante.fr](http://www.has-sante.fr)
14- HAS. Methodological guide: Structuring a therapeutic patient education program in the field of chronic diseases. HAS; 2007. Accessible on:[www.has-sante.fr](http://www.has-sante.fr)
15- Chauvigné C, Coulet JC. The skills-based approach: a new paradigm for university pedagogy? French Review of Pedagogy. 2010:15-28.
16- HAS: Therapeutic education of the patient. Definition, purposes and organizations. June 2007.
17- Sandrin-Berthon B. Educational diagnosis or shared educational assessment? Medicine of metabolic diseases, Edition Masson.2010;4(1):38-43.
18- Simon D, Traynard PY, Bourdillon, Grimaldi A. Therapeutic education: prevention and chronic diseases. Elsevier Masson ed, Issy-les Moulineaux 2007, 269 pages.
19- D'Ivernois JF, Gagnayre R. Learning to educate the patient. Maloine ed, Paris 2004, 155 pages.
20- The stages of the educational approach. Argument. Rev Mal Respir.2002; 19:2S59-2S75. SPLF, Paris 2002.
21- World Health Organization. Therapeutic patient education: continuing education programs for healthcare providers in the field of prevention of chronic diseases. Copenhagen: WHO; 1998.
22- National Institutes of Health, National Heart Lung and Blood Institute. Guidelines for the diagnosis and management of asthma. Bethesda (MD): NIH; 1997.
23- National Heart Lung and Blood Institute. World Health Organization. Beating Asthma: A Global Strategy for Asthma Management and Prevention. Rev FR Allergo Imminol Clin. 1996;(36)6.
24- Annual evaluation of a patient therapeutic education (TPE) program: a self-assessment approach. Methodological guide for coordinators. HAS. May 2014. Available on:[https://www.has-sante.fr/portail/jcms/c_1234324/fr/evaluation-annuelle-d-un-programme-d-education-therapeutique-du-patient-etp-une-demarche-d-auto- assessment](https://www.has-sante.fr/portail/jcms/c_1234324/fr/evaluation-annuelle-d-un-programme-d-education-therapeutique-du-patient-etp-une-demarche-d-auto-evaluation)
